# The inhibition of YAP Signaling Prevents Chronic Biliary Fibrosis in the Abcb4^-/-^ Model by Modulation of Hepatic Stellate Cell and Bile Duct Epithelium Cell Pathophysiology

**DOI:** 10.14336/AD.2023.0602

**Published:** 2024-02-01

**Authors:** Liangtao Ye, Andreas Ziesch, Julia S. Schneider, Andrea Ofner, Hanno Nieß, Gerald Denk, Simon Hohenester, Doris Mayr, Ujjwal M. Mahajan, Stefan Munker, Najib Ben Khaled, Ralf Wimmer, Alexander L. Gerbes, Julia Mayerle, Yulong He, Andreas Geier, Enrico N. De Toni, Changhua Zhang, Florian P. Reiter

**Affiliations:** ^1^Digestive Diseases Center, The Seventh Affiliated Hospital, Sun Yat-sen University, Shenzhen, China.; ^2^Department of Medicine II, University Hospital, LMU Munich, Germany.; ^3^Biobank of the Department of General, Visceral and Transplantion Surgery, University Hospital, LMU Munich, Germany.; ^4^Institute of Pathology, Faculty of Medicine, LMU Munich, Germany.; ^5^Division of Hepatology, Department of Medicine II, University Hospital Würzburg, Würzburg, Germany.

**Keywords:** Chronic biliary fibrosis, Primary sclerosing cholangitis, YAP, Mechanotransducer, Targeted therapy

## Abstract

Primary sclerosing cholangitis (PSC) represents a chronic liver disease characterized by poor prognosis and lacking causal treatment options. Yes-associated protein (YAP) functions as a critical mediator of fibrogenesis; however, its therapeutic potential in chronic biliary diseases such as PSC remains unestablished. The objective of this study is to elucidate the possible significance of YAP inhibition in biliary fibrosis by examining the pathophysiology of hepatic stellate cells (HSC) and biliary epithelial cells (BEC). Human liver tissue samples from PSC patients were analyzed to assess the expression of YAP/connective tissue growth factor (CTGF) relative to non-fibrotic control samples. The pathophysiological relevance of YAP/CTGF in HSC and BEC was investigated in primary human HSC (phHSC), LX-2, H69, and TFK-1 cell lines through siRNA or pharmacological inhibition utilizing verteporfin (VP) and metformin (MF). The Abcb4^-/-^ mouse model was employed to evaluate the protective effects of pharmacological YAP inhibition. Hanging droplet and 3D matrigel culture techniques were utilized to investigate YAP expression and activation status of phHSC under various physical conditions. YAP/CTGF upregulation was observed in PSC patients. Silencing YAP/CTGF led to inhibition of phHSC activation and reduced contractility of LX-2 cells, as well as suppression of epithelial-mesenchymal transition (EMT) in H69 cells and proliferation of TFK-1 cells. Pharmacological inhibition of YAP mitigated chronic liver fibrosis *in vivo* and diminished ductular reaction and EMT. YAP expression in phHSC was effectively modulated by altering extracellular stiffness, highlighting YAP's role as a mechanotransducer. In conclusion, YAP regulates the activation of HSC and EMT in BEC, thereby functioning as a checkpoint of fibrogenesis in chronic cholestasis. Both VP and MF demonstrate effectiveness as YAP inhibitors, capable of inhibiting biliary fibrosis. These findings suggest that VP and MF warrant further investigation as potential therapeutic options for the treatment of PSC.

## INTRODUCTION

Liver fibrosis consistently develops as a result of various forms of chronic liver diseases (CLD) [1]. Approximately 2% of the general population is impacted by liver fibrosis, and its incidence is anticipated to increase [2, 3]. Regardless of its etiology, liver fibrosis may ultimately progress to liver cirrhosis and its complications, which encompass the development of portal hypertension and/or hepatocellular carcinoma (HCC).

Hepatic stellate cells (HSC) represent a major source of extracellular matrix (ECM) in CLD [1]. The Hippo pathway, with its effector Yes-associated protein (YAP), has been identified as a central regulator of HSC activation and liver fibrosis in rodents [4, 5]. YAP acts as a key regulator of connective tissue growth factor (CTGF) expression, a central molecule implicated in the development of liver fibrosis. Notably, HSC serve as a major source of CTGF, which drives fibrosis development by inducing type I collagen expression [6]. Additionally, CTGF has been reported as a crucial player in biliary fibrosis [7]. The significance of the YAP downstream CTGF [4] as a potential therapeutic target for liver fibrosis has been demonstrated in several studies employing siRNA-based knockdown of CTGF, which resulted in prevention or inhibition of liver fibrosis in rodents [8, 9]. The crosstalk between YAP and the profibrotic player CTGF supports the hypothesis that YAP could be a potential target for treating liver fibrosis.

While YAP is vital for liver regeneration in acute liver damage [10], its inhibition by single- or short-term (3 weeks) injection of verteporfin (VP)-a well described YAP-inhibitor [4, 5, 11], resulted in reduced hepatic fibrosis in acute hepatitis induced by CCl_4_. Moreover, activation of YAP signaling is postulated as a central oncogenic driver in CLD [11, 12]. Despite these convincing results regarding the relevance of YAP protein in liver fibrogenesis upon hepatocellular damage, its role in chronic biliary fibrosis remains unclear. Enhanced nuclear expression of YAP has been described in various biliary diseases [12]. This finding is valuable since accumulating bile acids (BAs) can activate YAP, which potentially leads to carcinogenesis [11, 12].

**Figure 1.**
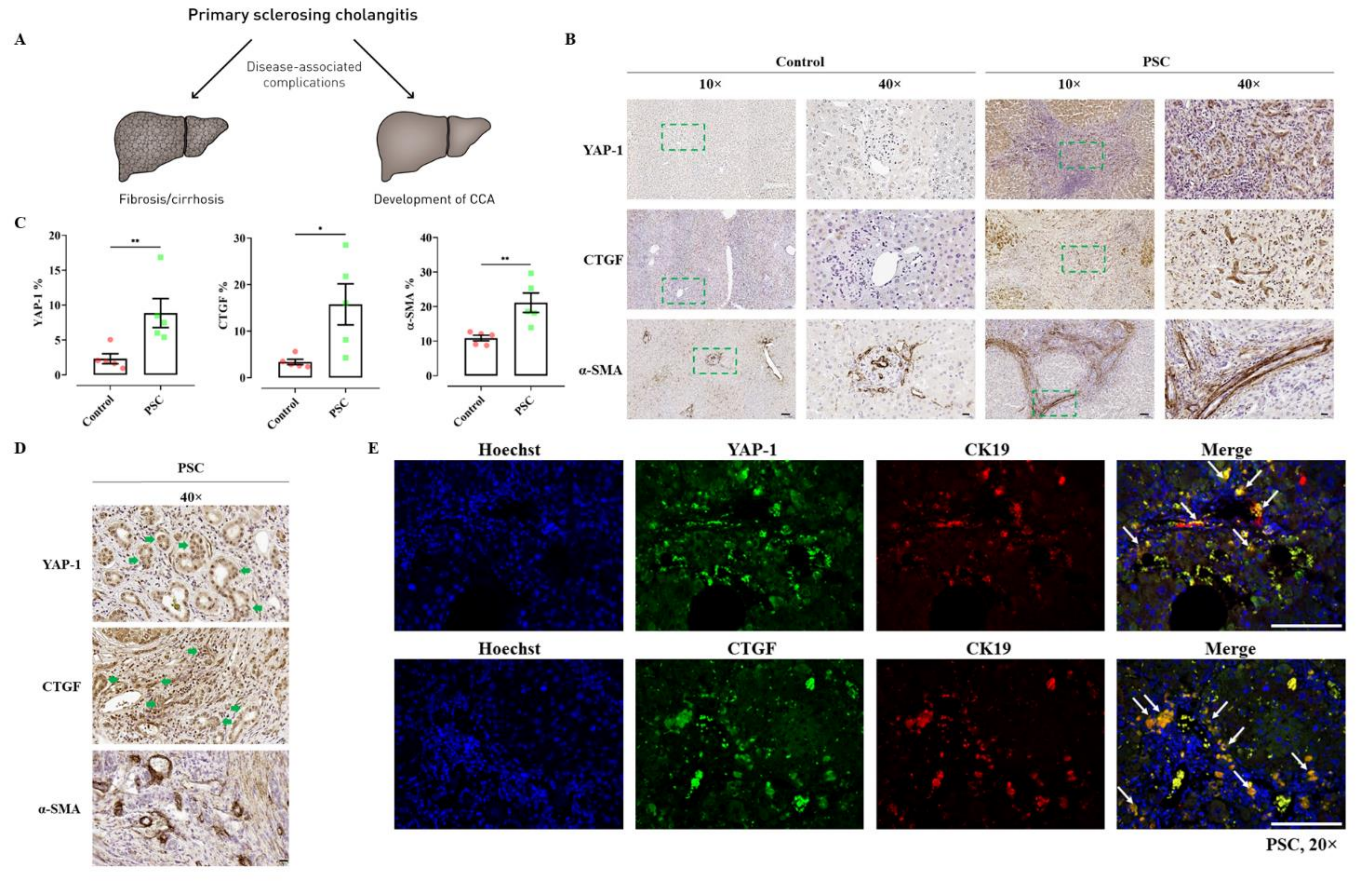
**Expression of YAP and CTGF in human liver tissues of PSC patients**. (A-B) Immunohistochemical staining of YAP, CTGF and α-SMA in human liver tissues of non-fibrotic control (samples obtained in non-tumoral liver areas) and PSC patients. Magnification, 10× (scale bar=100 µm) and 40× (scale bar=20 µm). Quantitative analysis of stained areas in percentage (%) was quantified by the QuPath software. n=5; *, *P*<0.05, **, *P*<0.01, *t*-test; test of normality by Shapiro-Wilk (*P*>0.05). The results are shown as mean ± standard error of the mean. The squares in dashed lines indicated the areas shown in 40-folds images. (**C**) Bile duct cells stained also positive for YAP and CTGF as illustrated by representative images. Magnification, 40× (scale bar=20 µm). (**D**) Representative immunofluorescence containing YAP (green)/CK19 (red) and CTGF (green)/CK19 (red) in liver tissues of PSC patients. Magnification, 20× (scale bar=100 µm).

Subsequent studies have demonstrated that the concurrent activation of YAP and AKT in hepatocytes leads to the development of cholangiocellular carcinoma (CCA) [11, 13], a tumor that is frequently observed in patients with primary sclerosing cholangitis (PSC) (Fig. 1A). This body of evidence raises the question of whether YAP inhibition might serve as a target for modulating liver injury in patients with chronic biliary liver diseases, such as PSC, where BAs are chronically elevated and effective medical treatments remain scarce [14]. However, the potential therapeutic effects of YAP inhibition on liver fibrosis and tumorigenesis are counterbalanced by its importance for liver regeneration. Genetic knockdown of YAP in a bile duct ligation (BDL) model resulted in acute liver necrosis due to impaired hepatocyte proliferation [15]. Nevertheless, acute and complete obstructive cholestasis by BDL [16] must be differentiated from liver regeneration in chronic cholestasis, where biliary flow is largely maintained without an increase in biliary pressure [17] (as demonstrated for ursodeoxycholic acid in the Abcb4^-/-^ model), and defective repair primarily occurs due to the accumulation of nonfunctional fibrotic tissues [16, 17]. Consequently, it remains a topic of debate whether YAP inhibition in chronic cholestasis is safe and effective enough to become a therapeutic target for treating PSC [18].

Although YAP inhibition has been reported to exhibit antifibrotic effects in multiple acute models of hepatocellular damage [4, 5, 15], its specific role in chronic biliary fibrosis, particularly in PSC, remains unclear. The present study aims to gain new insights into the role of Hippo signaling in biliary liver fibrosis, thereby providing a rationale for a novel and feasible pharmacological approach to ameliorate chronic cholestatic liver disease through YAP inhibition in PSC [11]. Moreover, this study strengthens the evidence supporting YAP as a central player in biliary fibrosis development by offering mechanistic insights into YAP's role in regulating the pathophysiology of HSC and bile duct epithelium cells (BEC). Lastly, this study further explores in-depth understanding of fibrogenesis perpetuation with respect to YAP as a mechanotransducer in HSC.

## MATERIAL AND METHODS

### Human liver samples

Double-coded tissues and corresponding data used in this study were provided by the Biobank of the Department of General, Visceral and Transplant Surgery, Ludwig-Maximilians-University (LMU). This Biobank operates under the administration of the Human Tissue and Cell Research (HTCR) Foundation. The framework of HTCR Foundation [19], which includes obtaining written informed consent from all donors, has been approved by the ethics commission of the Faculty of Medicine at the LMU (approval number: 025-12) as well as the Bavarian State Medical Association (approval number: 11142) in Germany. The use of all human materials presented in this paper was specifically approved by the ethical committee of the Faculty of Medicine at the LMU (project ID: 17-619).

### Immunohistochemistry

Paraffin-embedded sections (3 μm) of liver tissues were used for immunohistochemical staining. Anti-YAP-1 monoclonal mouse antibody (Abcam, UK; ab56701), anti-CTGF polyclonal rabbit antibody (Abcam, UK; ab6992), anti-α-SMA polyclonal rabbit antibody (Abcam, UK; ab5694), anti-collagen I monoclonal rabbit antibody (Abcam, UK; ab138492), anti-CK-19 monoclonal rabbit antibody (Abcam, UK; ab76539), and anti-S100A4 monoclonal rabbit antibody (Abcam, UK; ab197896) were applied as primary antibodies and detected by EnVision+System HRP-labeled polymer anti-mouse/rabbit antibodies (Dako, USA; K500711-2). Controls with only secondary antibody were employed to validate the specificity and distinguish non-specific binding in the background. The whole slides were scanned using the Pannoramic MIDI II^®^ digital slide scanner from 3DHistech (Sysmex, Germany). Quantitative analysis of stained areas was quantified and data was extracted objectively by the QuPath software (UK) [20]. QuPath is used for bioimage analysis of digital pathology applications with interactive machine learning both for object and pixel classification (https://qupath.github.io/).

### Immunofluorescence

Primary human hepatic stellate cells (phHSC) were incubated with anti-α-SMA polyclonal rabbit antibody (Abcam, UK; ab5694), anti-glial fibrillary acidic protein (GFAP) monoclonal mouse antibody (Sigma-Aldrich, Germany; G6171), anti-desmin polyclonal rabbit antibody (Sigma-Aldrich, Germany; SAB4500642), anti-YAP-1 monoclonal mouse antibody (Abcam, UK; ab56701), and anti-CTGF polyclonal rabbit antibody (Abcam, UK; ab6992), independently. H69 cells were incubated with anti-S100A4 monoclonal rabbit antibody (Abcam, UK; ab197896) and anti-E-cadherin monoclonal mouse antibody (Invitrogen, Germany; ECCD-2), independently. Human and mouse liver tissues were co-incubated with anti-CK-19 monoclonal mouse antibody (Proteintech, USA; 3G1E4) and anti-YAP-1 polyclonal rabbit antibody (Abcam, UK; ab62751), or anti-CTGF polyclonal rabbit antibody (Abcam, UK; ab6992). Secondary antibody was incubated with Alexa Fluor 488 goat anti-rabbit IgG (Invitrogen, Germany; AB_143165) and/or Alexa Fluor 594 goat anti-mouse IgG (Invitrogen, Germany; AB_2534073), as applicable. Controls with only secondary antibody were employed to validate the specificity and distinguish non-specific binding in the background. Nuclei were counterstained with Vectashield (Vector Laboratories, USA) containing Hoechst 33342 (Sigma-Aldrich, Germany). Pictures were taken using the Leica fluorescence microscope (Leica Microsystems, Germany).

### Abcb4^-/-^ mice

Male Abcb4^-/-^ (ATP-binding cassette, sub-family B, member 4) mice (FVB/N) were obtained from Jackson Laboratory (USA). The concentration of verteporfin (VP; Biorbyt, UK) and metformin (MF; Sigma-Aldrich, Germany) *in vitro* was determined by proliferation and WST-1 assays in HSC, while the concentration *in vivo* was referred to publications [4, 5, 21]. VP was diluted in 1% DMSO (ROTH, Germany) and 0.9% NaCl and was administered i.p. three times per week in low (50 mg/kg b.w.) or high (100 mg/kg b.w.) dosages. MF was diluted in 1% DMSO and 0.9% NaCl and was injected i.p. three times per week in low (35 mg/kg b.w.) or high (70 mg/kg b.w.) dosages. Abcb4^-/-^ mice were injected i.p. three times per week with low or high dosages of VP or MF (as mentioned above) beginning at 8 weeks of age, a time-point when the biliary fibrotic phenotype is fully established [16]. VP or MF was administrated for the following 12 weeks to stimulate long term treatment. Control mice (FVB/N wildtype) received vehicle injections consisting of 1% DMSO and 0.9% NaCl. All mice were housed in a 12/12 hours light/dark facility and were fed ad libitum with water and standard chow diet (ssniff, Germany). The mice were maintained according to the local regulations. All animals received human care, and the study protocols complied with the institution’s guidelines. All institutional and national guidelines for the care and use of laboratory animals were followed. The experiments were approved by the local authorities (Regierung von Oberbayern).

### Sirius red staining

Liver samples were fixed using 4% formaldehyde. After embedding in paraffin, 3-μm sections were stained with Sirius red according to the standard protocol. Slides were scanned using the Pannoramic MIDI II^®^ digital slide scanner from 3DHistech (Sysmex, Germany). The stained fibrotic area on the Sirius red-stained slides was quantified via QuPath software (UK) and ImageJ software (USA) as reported previously [22].

### Quantitative real-time PCR

RNA was isolated using peqGOLD TriFast (Peqlab, Germany) and complementary DNA was synthesized accordingly. Quantitative real-time PCR was performed in a SYBR^®^ Green system (QuantiTect SYBR Green PCR Kit, Qiagen, Netherlands) using a LightCycler 96^®^ (Roche, Germany). Expression was calculated according to the ΔΔCt method with GAPDH and 36B4 as the housekeeping genes and normalized to the means of the controls.

### Wildtype and Abcb4^-/-^ mice at 8 weeks

Mice liver tissues of FVB/N wild type and Abcb4^-/-^ (8 weeks of age) were used for analysis [23].

### GEO expression analysis

The microarray dataset GSE68001 and GSE4612 were downloaded from Gene Expression Omnibus (GEO; http://www.ncbi.nlm.nih.gov/geo) database. The dataset of GSE68001 (GPL13667 platform of Affymetrix Human Genome U219 Array) contained the transcriptomic profile of primary human quiescent HSC and *in vitro* activated HSC [24]. The GSE4612 dataset (GPL339 platform of Affymetrix Mouse Expression 430A Array) collected RNA samples of livers from control Abcb4^+/-^ and Abcb4^+/+^ mice at 3 and 12 months of age [25]. The *affy* package was applied for normalization of raw affymetrix data. Differential expression analysis was performed using *lmFit* and *eBayes* functions in *limma* package. *P* <0.05 adjusted by the false discovery rate (FDR) and and |log2 (fold change)|>1 were considered as significant.

### Cell culture of LX-2, H69, and TFK-1

LX-2 cells were purchased from Merck-Millipore (Germany). The authenticity of the LX-2 was analyzed by an external independent institution (Leibniz-Institut; DSMZ-Deutsche Sammlung von Mikroorganismen und Zellkulturen GmbH) and was classified as authentic according to the report of the institution. LX-2 cells were maintained in Dulbeccoʼs modified eagle medium (DMEM; Sigma-Aldrich, Germany) containing 2% fetal bovine serum (FBS; PAN-Biotech, Germany) and 1% antibiotics (Sigma-Aldrich, Germany) in a humidified atmosphere with 5% CO_2_ at 37 ^°^C. LX-2 cells were activated with 10 ng/ml of TGF-β (Peprotech, Germany).

The immortalized nonmalignant human bile duct epithelial cell line, H69 was cultured in DMEM/F12 (3:1)-based medium (Sigma-Aldrich, Germany) containing 10% FBS (PAN-Biotech, Germany) and 1% antibiotics (Sigma-Aldrich, Germany), enriched with 4 mM/L of glutamine (Sigma-Aldrich, Germany), 0.18 mM/L of adenine (Sigma-Aldrich, Germany), 5 mg/L of insulin (Sigma-Aldrich, Germany), 1.64 µM/L of epidermal growth factor (Sigma-Aldrich, Germany), 2 nM/L of triiodo-L-thyronine (Sigma-Aldrich, Germany), 1.1 µM/L of hydrocortisone (Pfizer, USA), 5.5 µM/L of epinephrine (Sigma-Aldrich, Germany), and 5 mg/L of transferrin (Sigma-Aldrich, Germany), and maintained in a humidified atmosphere with 5% CO_2_ at 37 ^°^C. The human bile duct carcinoma cell line TFK-1 was cultured in RPMI 1640 medium (Sigma-Aldrich, Germany) containing 10% FBS and 1% antibiotics, and maintained in a humidified atmosphere with 5% CO_2_ at 37 ^°^C.

### siRNA interference

LX-2 or H69 cells were plated to reach a confluence of 40%-60%. After overnight incubation, the cells were transfected using Oligofectamine (Invitrogen, Germany) and siRNA directed against YAP-1, CTGF, or with β-galactosidase (Dharmacon, UK) that served as control at a final concentration of 25 nM. Serum-containing medium was added 4 h after transfection. Silencing of YAP-1 or CTGF was confirmed by immunoblotting 24 h and 48 h after transfection. For siRNA transfection in phHSC, cells were cultured on uncovered plastic for 13 days. Transfection with the respective YAP-1-, CTGF-, and control-siRNA was performed on day 3, 7, and day 10.

### Immunoblotting

Cells were lysed in a standard protein lysis buffer. Protein concentrations were measured with Bradford reagent (Bio-Rad, Germany). Protein was loaded in equal amounts and separated by sodium dodecyl sulfate polyacrylamide gel electrophoresis, which followed by transferring to polyvinylidene difluoride membranes (Merck Millipore, Germany). Membranes were incubated with primary antibodies against YAP-1 (Abcam, UK; ab56701), CTGF (Abcam, UK; ab6992), α-SMA (Abcam, UK; ab5694), platelet-derived growth factor receptor-β (PDGFR-β; Cell Signaling Technology, USA; #3170), S100A4 (Abcam, UK; ab197896), Phospho-YAP-1 (Cell Signaling Technology, USA; #13008), Integrin αVβ6 (Bioss, USA; bs-5791R), TGF-β1 (Abcam, UK; ab215715), GAPDH (Abcam, UK; ab181602), Lamin B1 (Cell Signaling Technology, USA; #13435), and β-actin (Sigma-Aldrich, Germany; A2228), independently, which followed by the incubation with anti-mouse IgG-HRP (GE Healthcare UK Limited, UK; RPN4301) or anti-rabbit IgG-HRP (GE Healthcare UK Limited, UK; NA934) secondary antibodies. The bands were visualized by SuperSignal West Pico Chemiluminescent Substrate (Thermo Fisher Scientific, UK) and photographed with an image acquisition system of ECL ChemoCam Imager (Intas GmbH, Germany).

### Cell proliferation

1000 to 1500 cells of LX-2 were seeded in 96-well plates, cultured overnight, and then incubated in the presence of different concentrations of VP or MF. H69 cells were initially transfected with siRNA targeting YAP-1 (25 nM) and seeded as 2000 cells per well in 96-well plates. β-galactosidase (25 nM; Dharmacon, UK) transfection of H69 was used as control. TFK-1 cells were transfected with siRNA targeting YAP-1 (25 nM) or CTGF (25 nM) and seeded as 1200 cells per well in 96-well plates, with β-galactosidase (25 nM) transfection of TFK-1 as control. After 6 (H69) or 3 (TFK-1) days, cells were washed with PBS (Sigma-Aldrich, Germany) and underwent osmotic lysis in 100 μl ddH_2_O for 45 min at 37 ^°^C. 0.2% Sybr green (Lonza, USA) was added to each well, fluorescence was measured (Promega, USA) and proliferation index was calculated as a ratio to control samples. Relative percentage of control was shown as a ratio to the mean of control group.

### H&E staining

Paraffin-embedded sections (3 μm) of liver tissues from Abcb4^-/-^ mice were used for hematoxylin and eosin (H&E) staining. After stepwise deparaffinization and rehydration, slides were stained according to standard procedures.

### 3D cell culture of phHSC

1000 cells of phHSC were well mixed into reduced growth factor BME2 (Basement Membrane Extract, Type 2; Pathclear) and seeded on 24-well plates. After polymerization of BME2, classical culture medium was added to the cells with or without TGF-β (10 ng/ml). Cells were passaged by mechanical dissociation into small fragments by trituration using a plastic pipet with splitting medium of Iscove basal medium (Merck Biochrom, Germany) containing 1% HEPES (1M; Thermo Fisher Scientific, USA), 1% GlutaMAX Supplement (Thermo Fisher Scientific, USA), and 0.2% Primocin (InvivoGen, France), and transferred to fresh BME2 or plastic plates where indicated.

### Isolation and culture of phHSC

phHSC were isolated by the Biobank of the Department of General, Visceral and Transplant Surgery in LMU using a modified two-step collagenase perfusion procedure followed by low-speed centrifugation. The supernatant from the first centrifugation at 72× *g*, which contained the non-parenchymal cell (NPC) fraction, was then used for the isolation of phHSC using a three-layer density gradient [26]. phHSC were cultured in Iscove basal medium (Merck Biochrom, Germany) containing 1% sodium pyruvat (Merck Biochrom, Germany), 1% NEAA (non essential amino acids; Merck Biochrom, Germany), 1% L-glutamin (Sigma-Aldrich, Germany), 1% antibiotics (Sigma-Aldrich, Germany), and 10% fetal bovine serum (PAN-Biotech, Germany) in a humidified atmosphere with 5% CO_2_ at 37 ^°^C as described previously [27].

### Alamar blue assay

phHSC were seeded at a density of 1000 cells in 100 µl culture media {containing 20 µl matrigel/BME2 (Pathclear) in 3D culture} as triplicates onto 96 well-plates and were cultured for further analysis. 10 µl of AlamarBlue reagent (DAL1025; Invitrogen, Germany) per well was added to the culture medium at a final concentration of 10% (v/v). Blank control was established with medium and AlamarBlue solution of the same experimental concentration. Fluorescent signals (excitation 560 nm, emission 590 nm) were detected by the fluorescence plate reader (Promega, USA).

### Hanging drop method

Freshly isolated phHSC were purified and resuspended with complete Iscove basal medium as stated above to reach a cellular concentration of 2.5×10^6^/ml. Droplets of the cell suspension (30 μl) were placed onto the lids of 100 mm dishes (SARSTEDT, Germany), which were inverted over dishes containing 10 ml PBS. Hanging drop cultures were incubated and after sufficient sedimentation time, the resulting coherent 3D cellular aggregates were harvested for analysis.

### Hoechst/PI staining

Fluorometric Hoechst 33342 (Sigma-Aldrich, Germany) and propidium iodide (PI; Sigma-Aldrich, Germany) method was used to detect cell death in 3D culture [28]. phHSC in hanging drop, plastic plate, and matrigel/BME2 (Pathclear) cultures were stained with Hoechst and PI at a final concentration of 1% (v/v) each. Staining solution (dyes in PBS) was directly added to culture medium with a blank control. Cells were stained for 30 min in an incubator with 5% CO_2_ at 37 ^°^C. Subsequent analysis was performed on the plate reader (Promega, USA) and also the fluorescence microscopy (Leica Microsystems, Germany). Excitation and emission wavelengths were 535 nm and 617 nm for PI, and 361 nm and 486 nm for Hoechst, respectively. During the measurement, all wells were measured for PI fluorescence first and after a 30-s delay the measurement of Hoechst fluorescence was performed. Relative fluorescence unit (RFU) was recorded, and the signals of background was subtracted.

### Cellular fractionation

To separate cytoplasmic and nuclear proteins, cells were washed in PBS and suspended in hypertonic buffer (50 mM HEPES (pH 7.5), 10 mM KCl, 1.5 mM MgCl_2_, 1 mM EDTA, 1 mM Na_3_VO_4_, 50 mM NaF, 1 mM PMSF, 1 μg/ml of leupeptin). After being incubated on ice for 30 min, cells were sheared by being passed 25 times through a 26-gauge needle. The lysates were centrifuged at 800 *g* for 5 min at 4°C to obtain the cytosolic and membrane fraction (supernatant) and nuclear fraction (pellet). The nuclear pellet was washed three times with hypotonic buffer and resuspended in hypotonic buffer containing 1% Triton X-100 and 150 mM NaCl for 30 min on ice. The supernatant of lysed pellet, which has nuclear proteins, was isolated after centrifugation at 12,000 *g* for 20 min at 4°C.

### Collagen gel contraction assay

LX-2 cells were plated at a density of 200,000 cells per well in 6-well plates. The gel contraction assay was performed as described by Bell *et al* [29]. Cells were seeded in 1% collagen (Corning, USA). Thirty minutes after collagen polymerization, the gel was mobilized from the surface using a pipette tip (Rainin, Switzerland) and medium containing stimulative agents was added to the gels. After 24 h or 48h of incubation, the gel area was measured using ImageJ software (USA) and the ratio of the gel area to the total well area was calculated.

### Serum biochemistry

Alanine transaminase, alkaline phosphatase, and total bilirubin were analyzed using a respons^®^ 910 analyzer (Diasys, Germany).

### Hydroxyproline quantification

Hydroxyproline content of liver tissues was determined according to Edwards *et al* [30].

**Figure 2.**
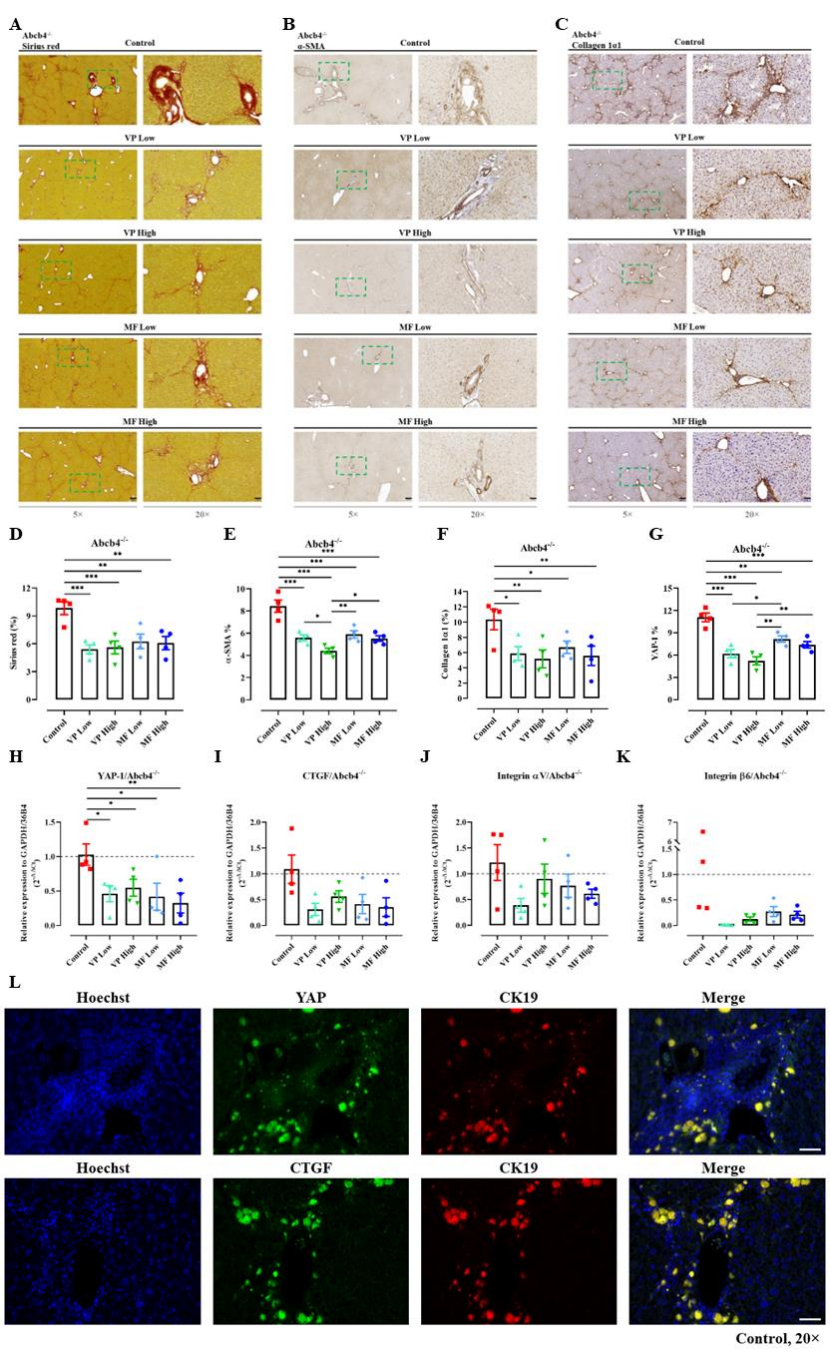
**Verteporfin and metformin prevent the progression of biliary fibrosis in the Abcb4^-/-^ model and downregulate the expression of YAP**. (**A**) The extent of fibrotic areas in sirius red-stained slides was analyzed quantitatively and representative images were shown (D). (B. C) Immunohistochemistry was performed to evaluate the positive area of α-SMA and collagen 1α1 in the liver. Representative images and quantitative analysis were shown (E. F). Magnification, 5× (scale bar=200 µm) and 20× (scale bar=50 µm). The squares in dashed lines indicated the areas shown in 20-folds images. (**G**) Immunohistochemical staining of YAP was performed in the liver tissues and analyzed quantitatively in percentage (%) by whole slide scanning. (H-K) The mRNA expression as 2^-∆∆Ct^ of YAP, CTGF, Integrin αV, and Integrin β6 under treatment was investigated in the liver. The values shown were normalized based on the mean of GAPDH and 36B4 in indicated control group. (**L**) Representative immunofluorescence co-staining of YAP/CK19 and CTGF/CK19 in control mice were shown. Magnification, 20× (scale bar=50 µm). VP, verteporfin; MF, metformin. Quantitative analysis of stained areas in percentage (%) was quantified by the QuPath software. n=4. *, *P*<0.05, **, *P*<0.01, ***, *P*<0.001, ANOVA; test of normality by Shapiro-Wilk (*P*>0.05). The results are shown as mean ± standard error of the mean.

### WST-1 cell viability assay (Water-soluble tetrazolium salt)

WST-1 assay was performed according to the manufacturer’s instructions (Roche, Germany).

### Statistical analysis

Data were analyzed by the SPSS version 26.0 (SPSS Inc., IBM, USA) or R (https://www.r-project.org). Statistical differences were assessed by Mann-Whitney U test, *t*-test, and analysis of variance (ANOVA) followed by the least significant difference (LSD) method for multiple groups, as applicable. Statistical significance was defined by *P* values <0.05. Results were shown as mean ± standard error of the mean of at least three independent experiments.

## RESULTS

### Dysregulation of the Hippo pathway in PSC patients

Previous studies have postulated the dysregulation of YAP in human liver fibrosis [4, 15]. The expression of YAP and its downstream gene CTGF were analyzed in human PSC samples by comparing liver tissues of PSC patients with those from non-fibrotic controls. Quantification of positively stained areas for YAP, CTGF, and alpha-smooth muscle actin (α-SMA) were performed using whole slide scanning. Significantly more positively stained areas for YAP and CTGF were observed in PSC samples compared to non-fibrotic controls (Fig. 1A-C; Supplementary Table 1). This finding aligned with higher counts of positively stained areas of α-SMA, indicating activation of myofibroblasts in fibrotic liver tissue (Fig. 1A-C). Interestingly, profound YAP and CTGF staining was also observed in cubic BEC by immunohistochemistry and co-staining of YAP/CTGF and CK19 (a marker for ductular reaction) (Fig. 1D-E), raising the question of whether YAP signaling is involved in BEC pathophysiology. In summary, these results demonstrate higher expression levels of YAP and CTGF in PSC patients.

### YAP mediates human HSC activation, and its pharmacological inhibition prevents liver damage and fibrosis

The significance of YAP as a player in fibrogenesis and HSC activation has been reported previously [4, 5, 11]. Through a comprehensive analysis, this study also confirmed YAP's involvement in the activation process and various profibrotic features (such as contractility) of human HSC (Supplementary Fig. 1) and demonstrated that its genetic and pharmacological inhibition can counteract TGF-β stimulation in human HSC (Supplementary Fig. 2-3). Metformin (MF), a compound known to exert beneficial effects in liver fibrosis in a pleiotropic manner [31], was also shown to inhibit YAP signaling *in vitro* and *in vivo* (Supplementary Fig. 2-6). For more details on these confirmatory experiments, please refer to the supplemental results section. Based on these findings, the role of YAP in the pathogenesis of cholestatic liver disease was investigated in a greater detail, using its specific inhibitor verteporfin (VP) to prevent biliary fibrosis in the Abcb4^-/-^ mice, which lack the ability to secrete phospholipids into the bile from the liver, representing a prototypical animal model of sclerosing cholangitis.

### Inhibition of the Hippo pathway reduces biliary fibrosis in Abcb4^-/-^ mice

Abcb4^-/-^ mice spontaneously develop sclerosing cholangitis, which resembles human PSC [32]. Since YAP inhibition has been described as potentially harmful in acute obstructive cholestasis due to its effects on liver regeneration, an initial assessment of potential negative effects in the Abcb4^-/-^ model of non-obstructive chronic cholestasis was conducted [15]. YAP signaling was inhibited by using two different dosages of VP or MF (Supplementary Fig. 4), and it was revealed that both compounds did not result in higher mortality over 12 weeks of treatment.

**Figure 3.**
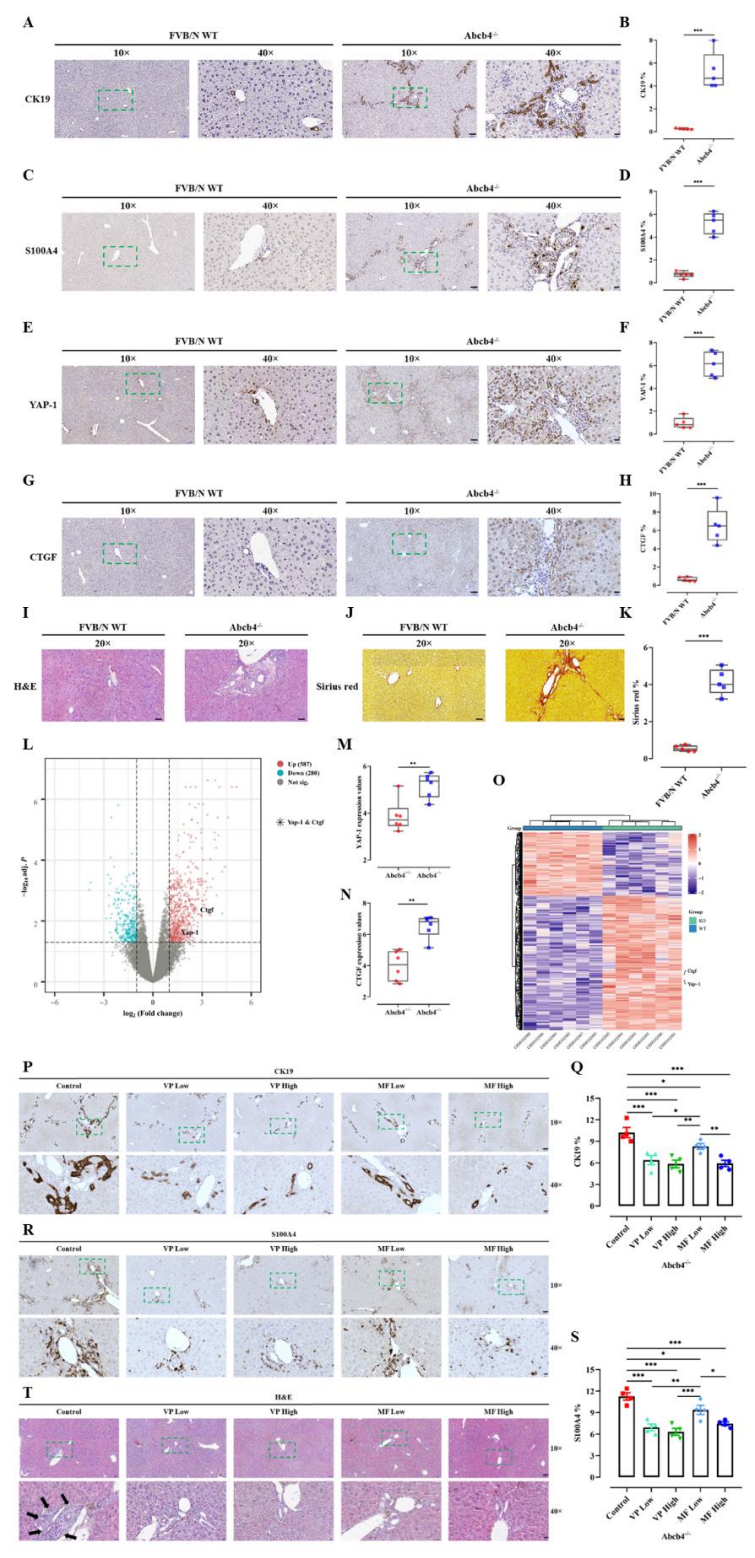
**YAP/CTGF is dysregulated in the Abcb4^-/-^ model and is associated with markers indicating ductular reaction and epithelial-mesenchymal transition (EMT), which could be ameliorated by verteporfin and metformin**. Immunohistochemical staining of CK19 (A), S100A4 (C), YAP (E), and CTGF (G) in liver tissues of Abcb4^-/-^ model and FVB/N wildtype (WT) control mice were performed. Magnification, 10× (scale bar=100 µm) and 40× (scale bar=20 µm). The squares in dashed lines indicated the areas shown in 40-folds images. Quantitative analysis of CK19 (B), S100A4 (D), YAP (F), and CTGF (H) stained areas in percentage (%) by whole slide scanning is shown. n=5. (I-J) H&E and sirius red staining were performed and quantitative analysis of the fibrotic areas was shown in percentage (%) (K). Magnification, 20× (scale bar=50 µm). n=5. (**L**) Volcano plot of Abcb4-heterozygotes (+/-) vs. Abcb4-KO (-/-) from the GSE4612 database was generated. Each dot represents a single gene. Horizontal axis: fold change (in log2 scale); vertical axis: adjusted *P*-value (in log10 scale). Upregulated genes are marked in red; downregulated genes are marked in blue. Dotted vertical lines highlight fold changes of -1 and +1, and the dotted horizontal line indicates *P*-value < 0.05. (M, N) Gene expression values of YAP and CTGF in comparison between Abcb4^+/-^ and Abcb4^-/-^ from GSE4612. n=6. **, *P*<0.01, ***, *P*<0.001, *t*-test; test of normality by Shapiro-Wilk (*P*>0.05). The results are shown as mean ± standard error of the mean. (**O**) Cluster analysis of differential gene expression was shown. (**P**) Immunohistochemistry was performed to stain the biliary epithelial cells (positive for CK19) in the liver. Representative images and quantitative analysis were shown (Q). (**R**) Immuno-histochemistry was performed to evaluate the number of cells undergoing EMT (positive for S100A4). Representative images and quantitative analysis were shown (S). Quantitative analysis of stained areas in percentage (%) was quantified by the QuPath software. (**T**) The treatments did not cause significant liver alterations when evaluated by H&E staining of liver (black arrows indicated biliary fibrosis). Magnification, 10× (scale bar=100 µm) and 40× (scale bar=20 µm). VP, verteporfin; MF, metformin. n=4. *, *P*<0.05, **, *P*<0.01, ANOVA; test of normality by Shapiro-Wilk (*P*>0.05). The results are shown as mean ± standard error of the mean. The squares in dashed lines indicated the areas shown in 40-folds images.

**Figure 4.**
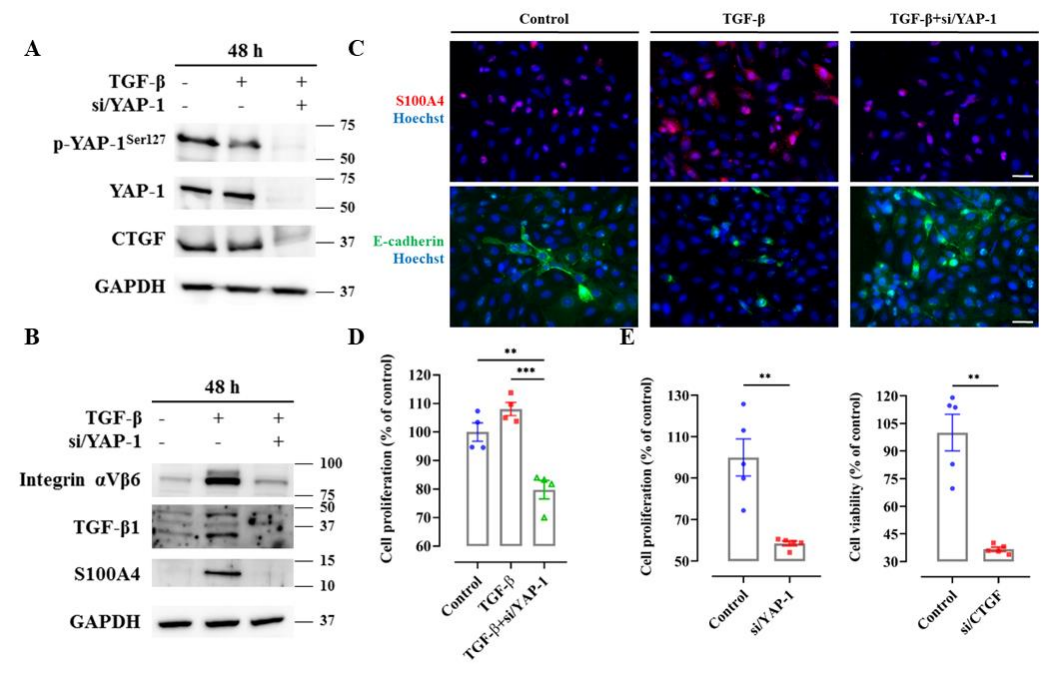
**Targeting YAP signaling in human biliary epithelial cells (H69) and in human bile duct carcinoma cells (TFK-1)**. (**A**) Protein expression of p-YAP, YAP, and CTGF was investigated upon TGF-β (5 ng/ml) stimulation for 48 hours after siRNA silencing of YAP in human H69 cells. These experiments were repeated three times independently. (**B**) siRNA targeting YAP inhibited upregulation of integrin αVβ6/TGF-β1 signaling and TGF-β mediated EMT in human H69 cells. These experiments were repeated three times independently. (**C**) Immunofluorescence staining of S100A4 and E-cadherin were shown in H69 cells under TGF-β stimulation. These experiments were repeated three times independently. Magnification, 20×, scale bar=50 µm. (**D**) Cell proliferation was assessed by Sybr green assay in H69 cells under TGF-β stimulation. n=4. The values were normalized to the mean of the control group in percentage (%). (**E**) Cell proliferation was assessed by Sybr green assay in TFK-1 cells after siRNA targeting YAP or CTGF. n=5. The values were normalized to the mean of the control group in percentage (%). **, *P*<0.01, *t*-test; test of normality by Shapiro-Wilk (*P*>0.05). The results are shown as mean ± standard error of the mean.

No mouse in the treatment group died within this observational period or needed to be sacrificed due to malaise. The physical phenotype of Abcb4^-/-^ mice was not negatively affected by the pharmacological inhibition of YAP, and treated mice displayed similar, or even higher (MF high group) body weights compared to control mice (Supplementary Fig. 5A-D).

Interestingly, both VP and MF reduced spleen/body weight compared to control mice (Supplementary Fig. 5E), suggesting their potential beneficial effects on portal hypertension. Additionally, stable liver parameters in biochemistry were observed in treated mice compared to untreated controls, indicating the feasibility of pharmacological YAP inhibition in chronic biliary fibrosis (Supplementary Fig. 5F-H). The anti-fibrotic effects of YAP inhibition, detected in non-cholestatic models of chronic liver damage, were confirmed in the Abcb4^-/-^ model of biliary fibrosis by reduced hydroxyproline levels (Supplementary Fig. 5I) and lower counts of sirius red-positive areas under VP and MF treatment (Fig. 2A). In accordance with these results, reduced α-SMA and collagen 1α1 protein expression was observed under MF and VP treatment, indicating their beneficial effects on HSC activation (Fig. 2B-F). Notably, VP and MF reduced YAP and CTGF mRNA expression, confirming their inhibitory effects on the activity of Hippo signaling (Fig. 2G-I). Furthermore, integrin αVβ6 mRNA expression, a postulated downstream of YAP signaling [5], was reduced, validating the functional inhibition of Hippo signaling (Fig. 2J-K). In summary, this study describes for the first time that inhibition of Hippo pathway could effectively reduce biliary fibrosis in Abcb4^-/-^ mice. In addition, no relevant side effects were observed from the continuous pharmacological inhibition, indicating an attractive new therapeutic target for modulating biliary fibrosis and PSC *in vivo*.

### YAP is involved in epithelial-mesenchymal transition and proliferation of human cholangiocytes and bile duct carcinoma cells

Cholangiocytes that undergo epithelial-mesenchymal transition (EMT) are postulated to be an important source of periportal myofibroblasts in biliary fibrosis [33, 34]. Initial experiments revealed strong YAP and CTGF expression in cubic BEC (Fig. 1D-E). This finding was confirmed by detecting strong co-expression of YAP and CK19-positive cholangiocytes in Abcb4^-/-^ mice (Fig. 2L).

Cholangiocytes are hypothesized to play a crucial role in the development of biliary fibrosis via EMT and ductular reaction [35], the role of Hippo signaling in this regard was investigated further. Liver tissue from wild-type (WT) and Abcb4^-/-^ mice was examined accordingly. Immunohistochemical staining of CK19 and S100A4 (a marker for EMT) was performed and analysed quantitatively by whole slide scanning (Fig. 3A-D). Both markers were elevated in Abcb4^-/-^ mice compared to WT controls, as expected. Interestingly, Abcb4^-/-^ mice also exhibited higher levels of YAP and CTGF expression (Fig. 3E-H). Furthermore, hematoxylin and eosin (H&E) staining demonstrated the histological characteristics of biliary fibrosis, with predominant periportal fibrotic areas indicated by sirius red staining in Abcb4^-/-^ mice (Fig. 3I-K). These findings were confirmed in an independent cohort by analyzing YAP and CTGF expression in Abcb4^+/-^ versus Abcb4^-/-^ mice using the open-source GSE4612 database (Fig. 3L-O). This result clearly confirmed the upregulation of Hippo signaling in this model of biliary fibrosis. Moreover, these effects aligned with results from *in vivo* studies, demonstrating lower expression of CK19 and S100A4 in Abcb4^-/-^ mice treated with VP or MF (Fig. 3P-T). While hepatic necrosis was observed under certain conditions by VP treatment [15], no relevant necrosis was detected in non-obstructive cholestasis under the investigated therapies of VP and MF (Fig. 3P-T).

As a subsequent step, experiments were conducted in a human cholangiocyte cell line (H69) to investigate the functional role of YAP by employing a siRNA-based knockdown. TGF-β, known to induce EMT in BEC such as H69 cells [34], was used to stimulate the H69 cells and induce EMT. It was verified that siRNA-based knockdown of YAP could lead to a reduction in YAP expression (Fig. 4A). A YAP knockdown resulted in diminished TGF-β-mediated S100A protein expression and preserved epithelial differentiation, as demonstrated by S100A4 and E-cadherin staining (Fig. 4B-C). Additionally, integrin αVβ6 protein expression was reduced as anticipated, validating the functional inhibition of Hippo signaling (Fig. 4B). The YAP knockdown also inhibited the proliferation of H69 cells under TGF-β stimulation (Fig. 4D), a cellular behavior potentially associated with profibrotic effects of BEC [36]. As we know, development of CCA represents a detrimental clinical event in patients with PSC. As a result, experiments were conducted in a cholangiocellular cell line (TFK-1) to assess the mechanistic relevance of YAP in oncogenesis. A notable reduction of proliferation, as measured by the Sybr Green assay, was observed upon siRNA-based YAP silencing in TFK-1 (Fig. 4E).

In summary, TGF-β-mediated EMT, as a potential profibrotic event in chronic cholangiopathy, is YAP-dependent. Furthermore, YAP mediates proliferation in cholangiocellular cancer cells. These findings strengthen the rationale of YAP inhibition as a therapeutic target for PSC.

### Extracellular stiffness induces HSC activation and correlates with nuclear translocation of YAP, indicating YAP as a mechanotransducer of HSC

YAP has been identified as a mechanotransducer in various organs, such as the liver, lung, and kidney [37]. As depicted in Figure 1B and 1C, levels of YAP and CTGF significantly increased in PSC, which raises a question of whether the activation of YAP and the increase of liver stiffness reflect a self-perpetuating vicious cycle in liver fibrogenesis. During the process of mechanotransduction and fibrogenesis, increased tension between the cells (HSC and BEC) and extracellular stiffness (ECM) leads to the induction of YAP tension-sensing pathway, which triggers a fibrotic response and, in turn, results in more collagen deposition and traction [38].

**Figure 5.**
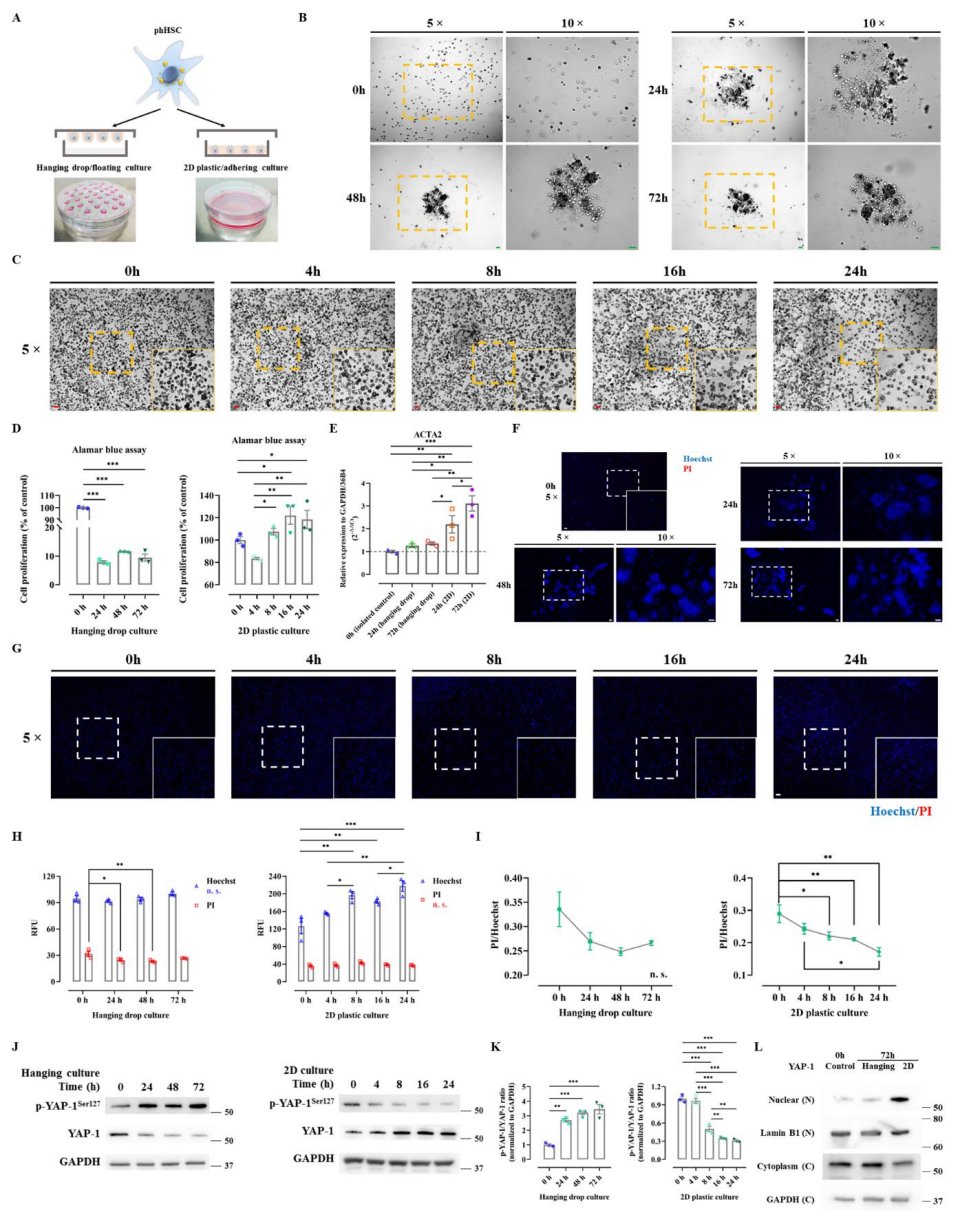
**The expression of YAP in phHSC is regulated by mechanosignaling when assessed by floating 3D hanging drop cultures compared to adhering 2D plastic cell cultures**. (**A**) Illustrating the principle of hanging drop cultures to reduce environmental stiffness in comparison to 2D culture. (B, C) Bright field images of hanging drop culture (B) and adhering 2D cultures (C) of phHSC after 0 h, 24 h, 48 h, and 72 h were illustrated. Magnification, 5× and 10×, scale bar=50 µm. (**D**) Alamar blue cell proliferation assay of hanging drop and 2D culture was indicated. n=3. The values were normalized to the mean of the control group in percentage (%).(E) Quantitative rt-PCR was performed to assess the expression of ACTA2 (2^-∆∆Ct^) in phHSC of hanging drop and 2D culture. (F, G) The pictures illustrated Hoechst/PI cell death staining of hanging drop culture (F) and of 2D culture (G) of phHSC. Magnification, 5×, scale bar=50 µm. (**H**) Relative fluorescence unit (RFU) of Hoechst (excitation 361 nm; emission 486 nm) and PI in hanging drop and 2D culture of phHSC. n=3. (**I**) PI and Hoechst ratio was calculated. n=3. (**J**) The protein expression of phospho (p)-YAP and YAP was assessed by western blot in hanging drop and 2D cultures of phHSC. The quantitative densitometry of p-YAP and YAP were normalized to baseline (GAPDH) and the ratio was calculated accordingly (K). (**L**) Protein expression of YAP in nuclear and cytoplasm was assessed by western blot in hanging drop and 2D cultures of phHSC in 72h. These experiments were repeated three times independently. n. s., not significant. *, *P*<0.05, **, *P*<0.01, ***, *P*<0.001, ANOVA; test of normality by Shapiro-Wilk (*P*>0.05). The results are shown as mean ± standard error of the mean. The squares in dashed lines indicated the areas shown in 10-folds images.

Initiation of HSC activation may be dependent on mechanosignaling from the extracellular microenvironment, which triggers the profibrotic nuclear transcription of YAP, promoting proliferation and trans-differentiation of HSC to myofibroblasts [4, 39]. Therefore, it was hypothesized that culturing HSC in 3D-hanging droplets, a technique that prevents cells from experiencing physical stiffness (nearly 0 pascal (Pa)), might prevent their activation. It is well documented that seeding HSC on plastic is sufficient for activation due to ultra-stiffness (1 gigapascal (Gpa); Fig. 5A) [4]. Freshly isolated phHSC were cultured in either floating or adhering conditions: after 24 hours of sedimentation, phHSC in droplets formed coherent 3D aggregates, while cells in conventional 2D adhered to the plastic surface (Fig. 5B-C). Alamar blue assay showed that growth of phHSC in floating status significantly decreased, while cells in 2D plates proliferated in a time-dependent manner (Fig. 5D). In this context, it was questioned whether the decreased proliferation in hanging droplets was related to the maintenance of quiescence status of phHSC or cell death. The mRNA expression of ACTA2 (α-SMA) in phHSC was maintained in hanging drop culture for 24 and 72 hours compared with freshly isolated phHSC (0 hour) (Fig. 5E).

However, the mRNA expression of ACTA2 significantly increased in 2D culture of phHSC at 24 hours and further increased until 72 hours in comparison with control and hanging droplets groups (Fig. 5E). Moreover, stainings for Hoechst33342 and propidium iodide (PI) were employed to detect dead cells by PI/Hoechst ratio [28]. Surprisingly, PI signals in hanging drop culture did not increase over time but slightly decreased, possibly related to cell division (Fig. 5F). As expected, 2D culture of phHSC led to increased Hoechst signals, potentially due to the fact that physical stimulation triggers activation and proliferation, showing increased Hoechst signal while PI intensity remained constant (Fig. 5G-H).

By normalizing dying or dead cells (PI-positive) to total DNA (Hoechst-positive), the ratio of quantified PI and Hoechst was calculated [28]. The PI/Hoechst signal gradually decreased, indicating that there was no significant proportion of cell death in both 3D-droplets and 2D over time (Fig. 5I). Interestingly, phHSC exhibited reduced proliferation in hanging drop culture without apparent cell death (Fig. 5H+I).

**Figure 6.**
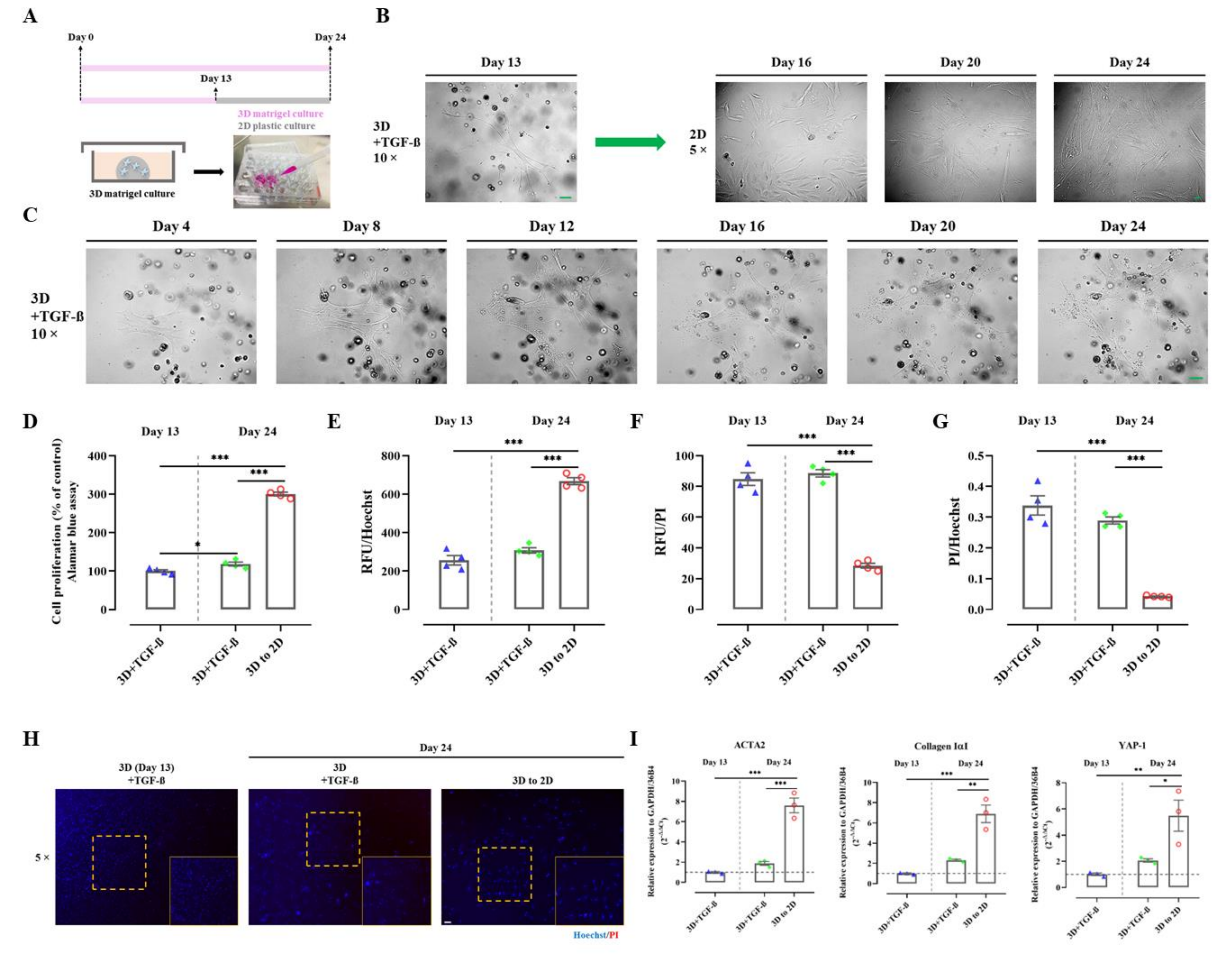
**Environmental stiffness mediates proliferation, activation, and YAP expression in phHSC**. (**A**) The figure illustrated the experimental design that intended to study the effects of transferring phHSC from 3D to 2D on day 13 of culture. As control groups used here: i. purely in 3D cultured phHSC; and ii. phHSC that were harvested on day 13 (the day of transferring from 3D to 2D). (**B**) Freshly isolated phHSC were cultured in 3D matrigel under stimulation with TGF-β (10 ng/ml) till day 13 and were then transferred to 2D plastic cultures and kept until day 24. Magnification, 5× and 10×, scale bar=50 µm. (**C**) Sequential images of phHSC cultured in 3D matrigel were illustrated. Magnification, 10×, scale bar=50 µm. (**D**) Alamar blue cell proliferation assays were performed. n=4. The values were normalized to the mean of the control group in percentage (%). Relative fluorescence unit (RFU) of Hoechst (excitation 361 nm; emission 486 nm) (E), PI (F), and PI/Hoechst ratio (G) of phHSC were measured. n=4. (**H**) The image illustrated representative Hoechst/PI staining of phHSC under different culture conditions. Magnification, 5×, scale bar=50 µm. (**I**) Quantitative rt-PCR was performed to assess the expression of ACTA2, collagen IαI, and YAP (2^-∆∆Ct^) in phHSC. n=3. The values shown were normalized based on the mean of GAPDH and 36B4 in indicated control group. *, *P*<0.05, **, *P*<0.01, ***, *P*<0.001, ANOVA; test of normality by Shapiro-Wilk (*P*>0.05). The results are shown as mean ± standard error of the mean. The squares in dashed lines indicated the areas shown in 10-folds images.

Consequently, it can be concluded that phHSC might maintain their quiescent, non-proliferative status simply due to the less-stiff microenvironment. This observation led to the hypothesis that YAP, as a mechanotransducer, might be involved. In this context, it was observed that the protein expression of p-YAP significantly increased after 24 hours when phHSC were cultured in hanging droplets, resulting in an elevated p-YAP/YAP ratio (Fig. 5J-K), indicating inactivity of YAP signaling. Conversely, phHSC seeded on 2D plates began to spread on the plastic surface after 4 hours, leading to the nuclear translocation of YAP and showing a reversed p-YAP/YAP ratio compared to cells in 3D-droplets (Fig. 5K). Furthermore, nuclear expression of YAP was significantly upregulated in 2D culture after 72 hours in comparison to phHSC in hanging droplets, which further validated that YAP nuclear translocation in phHSC could be regulated by physical stiffness mechanically (Fig. 5L). In summary, mechanotransduction induced by increased environmental stiffness mediates the proliferation of phHSC and triggers nuclear translocation of YAP and activation of Hippo signaling. This finding was further supported by using a different approach involving matrigel culture (see supplement).

### A physical switch from 3D to 2D activates HSC and upregulates YAP

The study demonstrated that transferring cells from a 'hard' to a 'soft' environment can prevent activation and proliferation of phHSC. For further investigation, phHSC were moved from matrigel to plastic (3D to 2D) at day 13 and harvested on day 24 (Fig. 6A). This was compared with cells kept in matrigel for 24 days and cells harvested at day 13 from matrigel (the point of transfer from 3D to 2D). The cells transferred to 2D developed a typical morphology of myofibroblasts, which was not observed in matrigel cultures (Fig. 6B-C). Alamar blue assay revealed that 3D cultures of phHSC displayed a slight increase in proliferation until day 24 compared with cells harvested on day 13 (Fig. 6D). However, the same batch of phHSC transferred to 2D exhibited significantly higher proliferation than both groups in 3D (Fig. 6D), which aligned with Hoechst signals (Fig. 6E). Notably, 3D cultures showed significantly higher intensity of PI signal and PI/Hoechst ratio (Fig. 6F-H), which might be related to a higher amount of cell fragments trapped in matrigel after isolation. Nonetheless, PI and PI/Hoechst did not increase over time in the 3D groups (day 13 vs. day 24) (Fig. 6F-H). Finally, rt-PCR confirmed that activation of phHSC and YAP mRNA expression increased due to the change in physical stiffness, as significantly higher mRNA expression levels of YAP, ACTA2, and Collagen IαI were detected upon transfer to 2D (Fig. 6I). These results confirm that the mechanical properties or stiffness of the extracellular matrix have profound effects on regulating the activation of HSC and YAP.

## DISCUSSION

**Figure 7.**
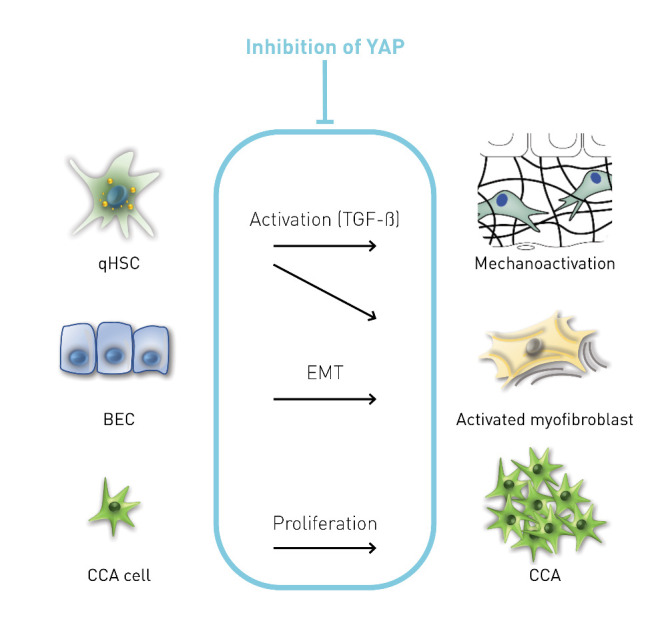
**YAP as a target for the treatment of PSC and its mechanism of action**. BEC, biliary epithelial cells; CCA, cholangiocellular carcinoma; EMT, epithelial-mesenchymal transition; qHSC, quiescent hepatic stellate cells.

PSC is a progressive CLD that frequently occurs in young adults. The poor prognosis of PSC is due to liver-related complications, including the development of liver cirrhosis or cancer, especially CCA. The etiopathogenesis of PSC is incompletely understood, and thus, no specific drug therapies are available to date. Liver transplantation is the only effective curative treatment, but PSC can reoccur even in transplanted livers [40]. Unfortunately, liver transplantation represents a very limited therapeutic resource due to organ shortage [41]. These facts underscore the high demand for effective therapies capable of preventing or delaying the progression of PSC. It is hypothesized that the YAP pathway may represent a potential molecular target for treatment of PSC due to the following reasons (Fig. 7):

- YAP is an important mechanoregulator of liver fibrogenesis and activator of HSC into myofibroblast [4, 5];
- YAP promotes EMT of BEC and development of CCA in the liver [11, 13];
- bile salts, including cholic acid (CA), chenodeoxycholic acid (CDCA), taurodeoxycholic acid (TDCA), and deoxycholic acid (DCA), which accumulate in cholestatic liver diseases such as PSC, activate YAP signaling [11, 12, 42-44].

However, YAP as a therapeutic target in liver disease is discussed controversially due to the fact that clinically tolerable pharmacological inhibitors are lacking, and harmful effects were reported for the BDL model of acute and complete obstructive cholestasis [11, 15]. Nevertheless, due to the well-described anti-fibrotic effects of YAP inhibition, it appears plausible to investigate YAP as a potential target modulating biliary fibrosis in the chronic cholestatic Abcb4^-/-^ model.

Aside from VP as an established YAP inhibitor, MF was explored as an alternative active inhibitor of YAP signaling for a more feasible and tolerable pharmacological approach superior to VP in YAP inhibition. The concentration of VP and MF *in vitro* was determined by proliferation and WST-1 assays in HSC (Supplementary Fig. 7), while the concentration *in vivo* was referred to publications [4, 5, 21]. It was revealed that comparable results between MF and VP could be observed in preclinical *in vitro* and *in vivo* fibrosis models. For this purpose, a non-obstructive model of cholestasis, the Abcb4^-/-^ model resembling sclerosing cholangitis, was utilized. The results strongly argue for MF unfolding its effects (besides several pleiotropic mechanisms of action) also by inhibition of YAP signaling. In this context, its employment resulted in an amelioration of liver fibrosis in the cholestatic Abcb4^-/-^ model (Fig. 2) [21]. Furthermore, inhibitory effects on HSC activation and profibrotic cell behavior were demonstrated in different *in vitro* models.

As mentioned previously, YAP inhibition can exhibit harmful effects in cholestasis under conditions of complete or subtotal obstruction. However, by utilizing a model with minor obstructive changes of the bile ducts, long-term pharmacological inhibition of YAP by VP or MF did not result in relevant side effects concerning liver function, which assessed by liver serum parameters or animal mortality (Supplementary Fig. 5). These findings suggest the translational potential of pharmacological YAP inhibition to modulate liver fibrosis in CLD, such as PSC. Moreover, to the best of current knowledge, it is shown for the first time that inhibition of YAP signaling prevents biliary fibrosis in Abcb4^-/-^ mice-a model mimicking PSC. These results could prompt a discussion on whether MF should be clinically investigated in PSC in future studies. In the study, the effects of MF and VP in WT mice and their potential effects on a reversal of HSC activation and fibrosis were not investigated, which might be of interest from a translational perspective and should be performed in future studies before this approach can be translated into human situation.

HSC activation is the major source for both biliary and non-biliary fibrosis [39]. However, initial fibrosis in chronic cholestatic liver disorders such as PSC demonstrates a particular pattern of fibrosis affecting primary portal tracts, resulting in pronounced portal to portal fibrosis, defined by the term biliary fibrosis [45]. This pattern may arise from the initial location of damage inside the portal fields in chronic cholestasis involving BEC [45]. BEC forming the space of Disse play a crucial role in the pathogenesis of biliary fibrosis [46], a fact that may differ from non-biliary fibrosis [45]. Chronic cholestasis leads to bile duct injury, resulting in damage to surrounding hepatocytes [47]. This damage, associated with the proliferation of BEC to form new biliary-type structures intending to resolve the damage, is called ductular reaction and coincides with an accumulation of myofibroblasts and ECM [45]. Consequently, the inhibition of both proliferation and EMT of BEC has been postulated as a potential target capable of ameliorating biliary fibrosis in chronic cholestatic disorders such as PSC [48].

It was further hypothesized that YAP is involved in EMT of BEC. The fact that YAP inhibition resulted in reduced EMT in BEC suggests that the effects on biliary fibrosis mediated by YAP inhibition may not only be related to modulation of HSC, but also to modulation of BEC pathophysiology (Fig. 7). Notably, YAP inhibition by siRNA led to a pronounced inhibition of proliferation in the CCA cell line (TFK-1). Whether these results may even indicate a potential therapeutic anti-tumor effect of YAP inhibition on CCA emergence or proliferation should be addressed in future studies (Fig. 7).

In this study, new mechanistic insights were gained into how YAP regulates HSC activation through mechanotransduction (Fig. 7). This may generally become relevant in liver fibrosis/cirrhosis when stiffness increases significantly due to the accumulation of ECM, potentially resulting in fibrosis development by self-perpetuating and progressing despite the elimination of underlying diseases [38]. In cholestatic diseases with increasing biliary pressure over time, this mechanism becomes even more crucial, as it is well established that even partial biliary obstruction increases liver stiffness in human elastometry studies [49]. In particular, this rationale led to the investigation of a potential application of mechanotherapy by targeting YAP to block the 'vicious loop' of fibrosis progression. In this work, the mechanical regulation is elucidated by establishing three physical culturing methods for phHSC, including floating droplets (non-attachment), 3D matrigel (soft substrate), and 2D plate (super stiffness). It is confirmed that extracellular stiffness *per se* directly impacts YAP activity and proliferation of HSC, depending on the degree of increased stiffness. These data demonstrate the importance of extracellular stiffness on HSC pathophysiology and reveal that the YAP pathway is regulated by stiffness in phHSC.

In summary, this study provides significant new rationale suggesting that YAP signaling may be an attractive target to modulate fibrosis (and potentially carcinogenesis) in chronic cholestatic liver disease, particularly in PSC (Fig. 7). Furthermore, novel mechanisms of therapeutic potential are presented by inhibiting YAP in HSC (mechanotransduction) and BEC (EMT) as crucial drivers of biliary fibrosis. Future studies are warranted to translate these findings into clinical application, particularly in chronic cholestatic diseases like PSC where no causal treatment is available to date.

## Supplementary Materials

The Supplementary data can be found online at: www.aginganddisease.org/EN/10.14336/AD.2023.0602.


