# Supplementary material for: The inhibition of YAP Signaling Prevents Chronic Biliary Fibrosis in the Abcb4-/- Model by Modulation of Hepatic Stellate Cell and Bile Duct Epithelium Cell Pathophysiology
Source: Aging Dis. 2024 Feb 1;15(1):338–56. doi: 10.14336/AD.2023.0602 (PMC10796084; doi:10.14336/AD.2023.0602)
Supplement: Supplementary file 1 [file AD-15-1-338-s.pdf]

SUPPLEMENTARY DATA

# The inhibition of YAP Signaling Prevents Chronic Biliary Fibrosis in the Abcb4$^{-/-}$ Model by Modulation of Hepatic Stellate Cell and Bile Duct Epithelium Cell Pathophysiology

**Liangtao Ye, Andreas Ziesch, Julia S. Schneider, Andrea Ofner, Hanno Nieß, Gerald Denk, Simon Hohenester, Doris Mayr, Ujjwal M. Mahajan, Stefan Munker, Najib Ben Khaled, Ralf Wimmer, Alexander L. Gerbes, Julia Mayerle, Yulong He, Andreas Geier, Enrico N. De Toni, Changhua Zhang, Florian P. Reiter**

© 2023. Ye L et al. Published online at http://www.aginganddisease.org/EN/10.14336/AD.2023.0602

# SUPPLEMENTARY DATA

## SUPPLEMENTARY RESULTS

### Expression of Hippo pathway members increase during activation of phHSC

Under chronic pathological stimuli, HSC transdifferentiate to activated myofibroblasts (activated HSC), which become a major source of ECM in liver fibrosis [50]. YAP has been demonstrated to be involved in the activation process of murine HSC [4, 5], while its role in human HSC has not been systematically investigated. phHSC were plated on uncovered plastic and underwent spontaneous activation, as evidenced by immunofluorescence and western blot (Suppl. Fig. 1A, B). While CTGF protein expression correlated in a time-dependent manner with that of fibrotic markers as α-SMA and PDGFR-β, the expression of YAP initially increased with a peak on day 11. The fact that CTGF, as the downstream gene of YAP and an indicator of YAP activation, increased synchronously with α-SMA and PDGFR-β may imply that the Hippo pathway participates in the HSC activation process and suggests a mechanistic role for YAP in the pathophysiology of human HSC. This finding was confirmed in a different human cohort through the analysis of microarray data from the Gene Expression Omnibus dataset (Suppl. Fig. 1C-F), which validated that both YAP and CTGF were elevated in activated HSC compared to quiescent HSC.

### Inhibition of YAP and CTGF by siRNA, VP, and MF impedes activation of phHSC and regulates TGF-β signaling in LX-2 cells

In the subsequent step, *in vitro* experiments were conducted in phHSC to examine whether inhibiting YAP and CTGF could reduce HSC activation in order to obtain mechanistic insights regarding their functional roles in the activation process of HSC. Consequently, a siRNA-based knockdown of YAP and CTGF was performed in phHSC. The efficacy of the knockdown was validated by western blot and immunofluorescence, revealing reduced YAP protein expression upon siYAP and diminished CTGF protein expression upon siCTGF (Suppl. Fig. 2A+C). The knockdown of both molecules led to decreased protein expression of α-SMA and PDGFR-β (Suppl. Fig. 2A+D), which confirmed the functional role of YAP and CTGF in HSC activation. Moreover, pharmacological inhibition was conducted by using the specific YAP inhibitor VP and the pleiotropic inhibitor MF. Both VP and MF displayed inhibitory effects on CTGF protein expression and phHSC activation (Suppl. Fig. 2B+E+F), suggesting MF as a potential clinically viable inhibitor of this pathway. To gain further mechanistic insights, inhibition of YAP and CTGF in LX-2 cells under TGF-β activation was examined. As a result, increased CTGF protein expression (Suppl. Fig. 3A) accompanied with decreased phospho-YAP expression (Suppl. Fig. 3B) was observed, indicating that Hippo pathway could be activated by TGF-β. Thus, the siRNA-knockdown of YAP not only resulted in lower YAP and CTGF protein expression but also prevented TGF-β-mediated activation, as evidenced by reduced α-SMA and PDGFR-β expression (Suppl. Fig. 3A). In addition, these effects were replicated by siRNA knockdown of CTGF (Suppl. Fig. 3A). These findings confirmed that the YAP pathway is mechanistically involved in TGF-β mediated activation of HSC. Interestingly, similar effects were achieved through pharmacological inhibition by VP or MF *in vitro* (Suppl. Fig. 3A). Furthermore, experiments on functional aspects of HSC physiology were performed by a gel contraction assay to explore whether YAP is involved in TGF-β-mediated contractility of HSC, and whether it represents a target for modulating portal hypertension in end-stage liver disease. As anticipated and as previously demonstrated [27], TGF-β induced contractility in LX-2 cells as depicted by a smaller collagen gel area (Suppl. Fig. 3C+D). In the study, inhibition of YAP and CTGF could effectively reduce the contractility after 24h and 48h using siYAP, siCTGF, VP, and MF, respectively (Suppl. Fig. 3C+D).

### Culturing HSC in 3D matrigel prevents HSC activation and inhibits YAP

To emphasize the impact of physical environment in HSC activation, experiments were extended to 3D matrigel culture (approximately 200-500 Pa of stiffness [51]). 3D culture mimics a tissue-like structure that closely resembles human physiology, potentially improving the predictive value of preclinical models, in contrast to traditional 2D culture where cells are grown in a flat monolayer [52]. This technique was employed to study the physiology of phHSC through a dynamic switch from a 'hard' to a 'soft' surface (Suppl. Fig. 6A), with conventional 2D plastic culture serving as a positive control. In 2D culture, phHSC completely transformed into myofibroblasts after 24 days, as indicated by a typical myofibroblast-like morphology (Suppl. Fig. 6B) and positive staining of fibrotic markers including α-SMA, desmin, and GFAP (Suppl. Fig. 6C). Additionally, phHSC were cultured for 3, 7, or 13 days in 2D plastic before being transferred to 3D matrigel, where the cells were maintained until day 24 as indicated {3D (day 3), 3D (day 7), and 3D

© 2023. Ye L et al. Published online at http://www.aginganddisease.org/EN/10.14336/AD.2023.0602

# SUPPLEMENTARY DATA

(day 13)} (Suppl. Fig. 6D-F). The goal was to study the impact of stiffness on phHSC behavior at different activation levels (day 3 quiescent; day 13 activated). Sequential images were recorded to monitor the growth patterns of phHSC in 3D, both with and without TGF-β (Suppl. Fig. 6D-F). It was found that cells in matrigel did not undergo significant cell death in long-term culture, as PI signals remained at basal levels in 3D compared to those in 2D (Suppl. Fig. 6G-K). The 3D groups displayed significantly lower Hoechst intensity compared to 2D groups on day 24 (the same batch of phHSC). Furthermore, higher PI/Hoechst ratios were measured in phHSC that were transferred to matrigel, with statistical significance in the 3D (13 day) group (Suppl. Fig. 6L), which potentially related to the decreased proportion of total DNA in 3D compared to 2D culture, as PI signals themselves did not reveal differences between the groups (Suppl. Fig. 6K). Alamar blue assays indicated that cell proliferation significantly decreased once phHSC were transferred from 2D plates to 3D matrigel, even on day 13 with activated phHSC (Suppl. Fig. 6M). Interestingly, TGF-β partially reversed these effects, demonstrating that cells in 3D maintained their potential to proliferate (Suppl. Fig. 6M). Rt-PCR revealed that mRNA expression of ACTA2, which codes for the α-SMA protein, was significantly lower in 3D culture when cells were transferred at early time points {3D (day 3) or 3D (day 7)}. This finding aligned with reduced YAP mRNA expression in all 3D groups compared to 2D (Suppl. Fig. 6N). It can be concluded that environmental stiffness is a potent inducer of phHSC activation and proliferation, and YAP is regulated by mechanical stimuli in phHSC.

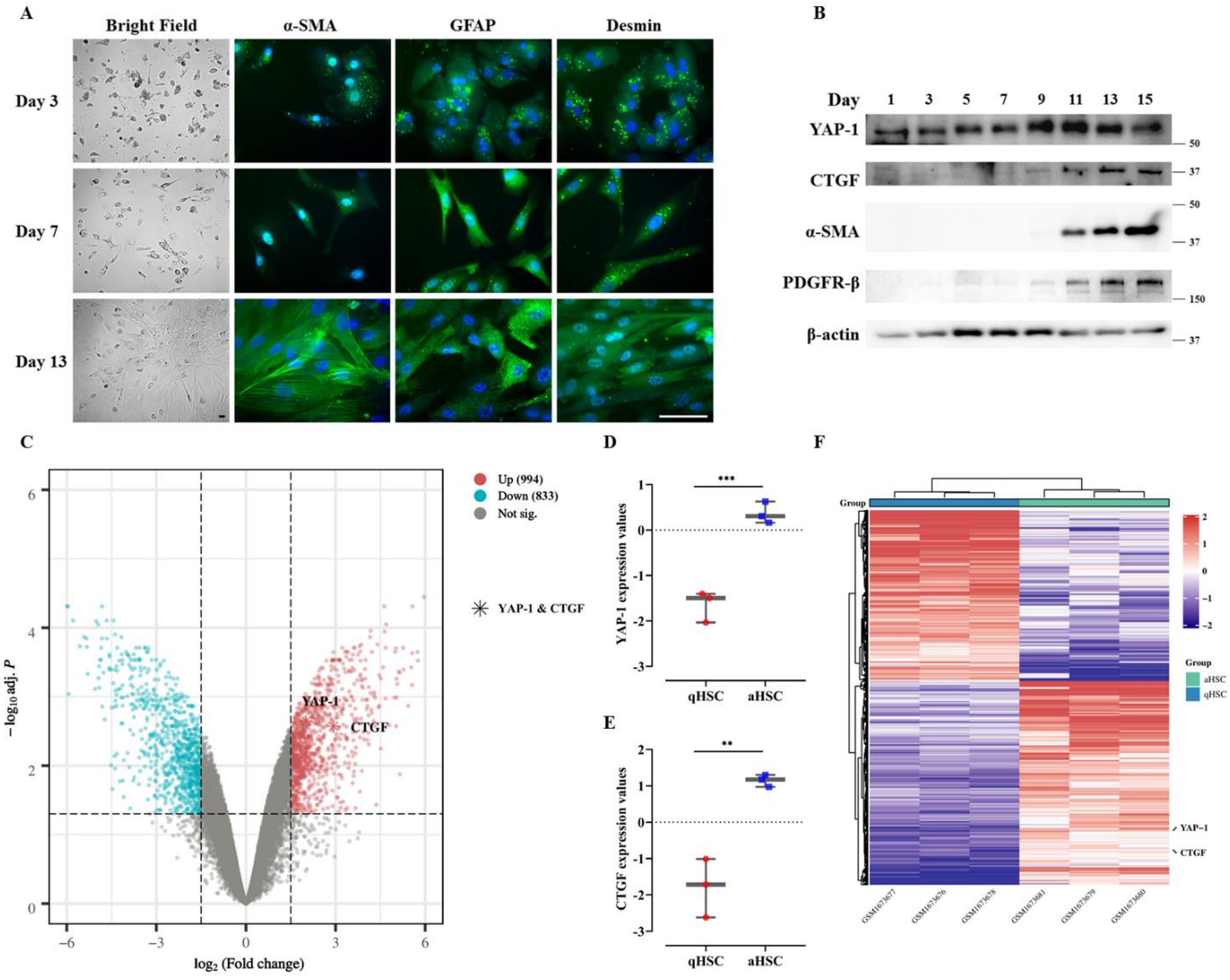


© 2023. Ye L et al. Published online at http://www.aginganddisease.org/EN/10.14336/AD.2023.0602

# SUPPLEMENTARY DATA

**Supplementary Figure 1. Expression of YAP and CTGF during *in vitro* cultivation of phHSC. A.** Immunofluorescence (IF) staining of the activation process of phHSC *in vitro*. Magnification, 5× (bright field) and 40× (IF), scale bar=50 μm. **B.** Representative western blots of three experiments for YAP and CTGF expression during the activation process of phHSC were shown. n=3. **C.** Volcano plot of quiescent (q) vs. activated (a) phHSC from the GSE68001 database was illustrated. Each dot represents a single gene. Horizontal axis: fold change (in log2 scale); vertical axis: adjusted *P*-value (in log10 scale). Upregulated genes are marked in red; downregulated genes are marked in blue. Dotted vertical lines highlight fold changes of -1 and +1, and the dotted horizontal line indicates $P$-value < 0.05. **D. E.** Gene expression values of YAP and CTGF comparing HSC with aHSC from GSE68001 were calculated. n=3. **, $P$<0.01, ***, $P$<0.001, *t*-test; test of normality by Shapiro-Wilk ($P$>0.05). The results are shown as mean ± standard error of the mean. **F.** Cluster analysis of differential gene expression was shown.

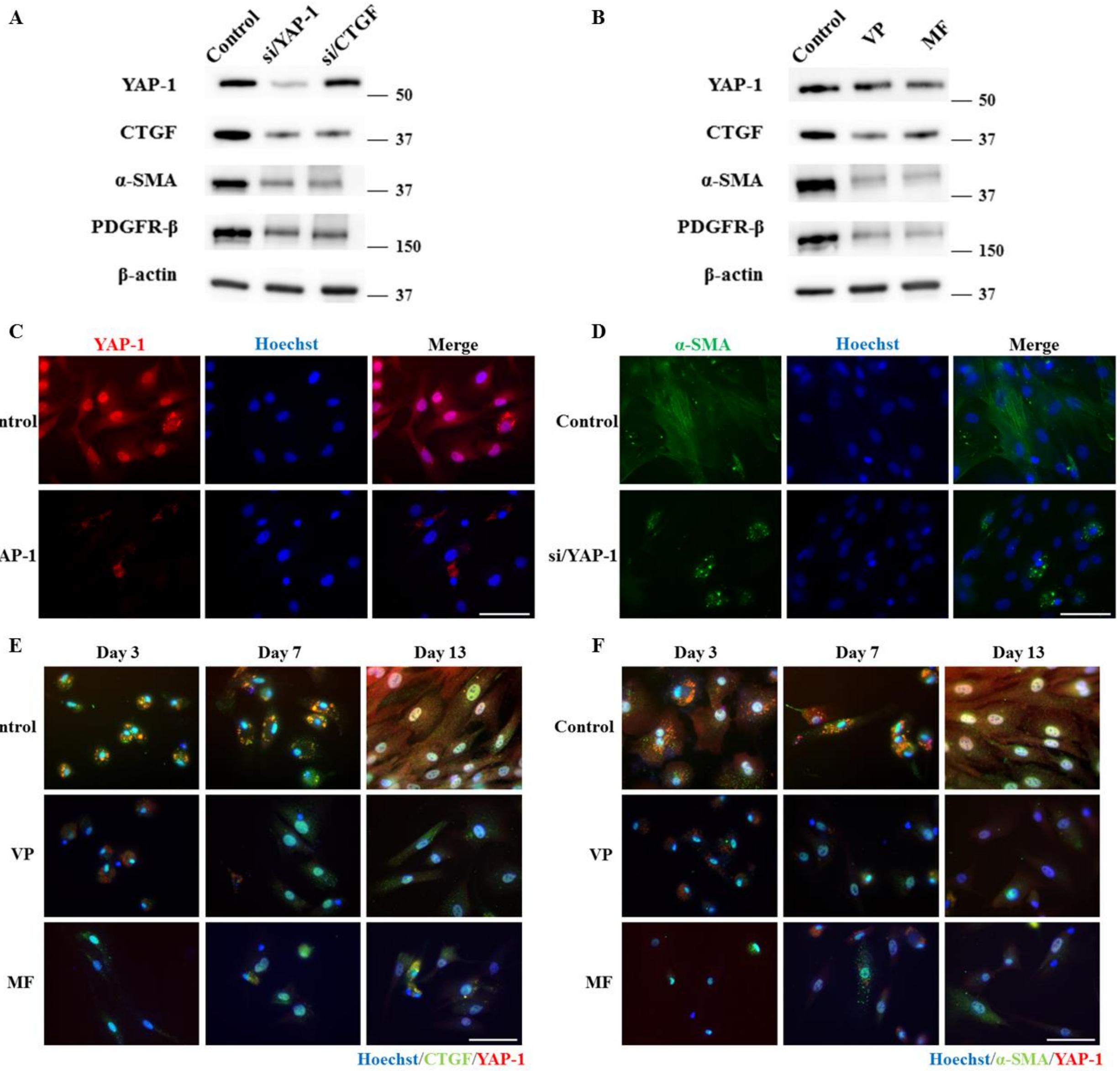


**Supplementary Figure 2. Inhibition of YAP and CTGF reduces spontaneous activation of phHSC *in vitro*. A. B.** Protein expression of YAP, CTGF, and markers indicating HSC activation as α-SMA and PDGFR-β were assessed by western blot under siRNA silencing of YAP and CTGF or targeted therapy with verteporfin (VP) and metformin (MF) on day 13. Representative blots of three experiments were illustrated. Immunofluorescence (IF) staining YAP **(C)** and α-SMA **(D)** under gene silencing using siRNA against YAP is shown on day 13 of phHSC. **E. F.** IF staining of YAP/CTGF and YAP/α-SMA under targeted therapy with VP or MF was illustrated on day 3, 7, and 13. Magnification, 40×, scale bar=50 μm. These experiments were repeated three times independently.

© 2023. Ye L et al. Published online at http://www.aginganddisease.org/EN/10.14336/AD.2023.0602

SUPPLEMENTARY DATA

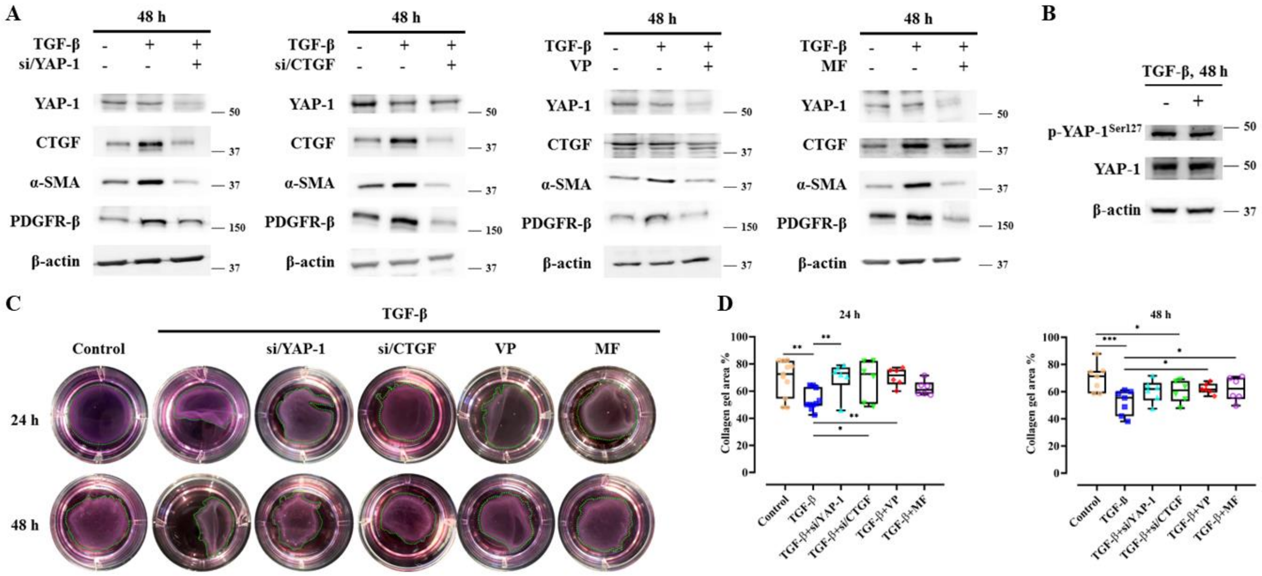


**Supplementary Figure 3. Effects of siRNA silencing or pharmacological inhibition of YAP and CTGF on TGF-β mediated profibrotic behavior in human LX-2 cells.** Human LX-2 cells were activated using 10 ng/ml transforming growth factor-β (TGF-β). Profibrotic behavior with TGF-β stimulation under siRNA-mediated silencing of YAP or CTGF, and under clinically viable YAP inhibitors verteporfin (VP; 1 µM) and metformin (MF; 2 mM) were investigated, independently. **A.** Protein expression of YAP, CTGF, and fibrogenic markers as α-SMA and PDGFR-β were studied by western blot. The experiments were repeated three times independently. **B.** Protein expression of phospho (p)-YAP and YAP were investigated upon TGF-β stimulation. n=3. **C. D.** Collagen gel contraction assays were performed to study the inhibitory effects on TGF-β-mediated contractility in human LX-2 cells. The gel areas were calculated as percentages (%) of the initial well-area and representative images after stimulation were shown. n=6. *, $P$<0.05, **, $P$<0.01, ***, $P$<0.001, ANOVA; test of normality by Shapiro-Wilk ($P$>0.05). The results are shown as mean ± standard error of the mean.

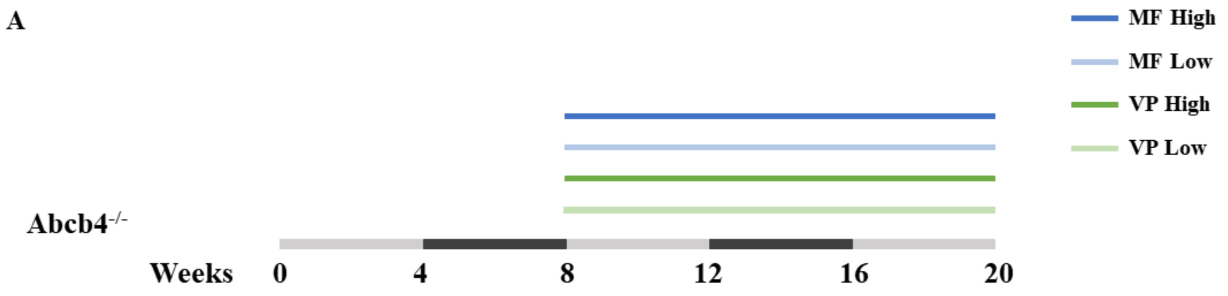


**Supplementary Figure 4. Study design *in vivo*. A.** Abcb4$^{-/-}$ mice (FVB/N) spontaneously develop biliary fibrosis and sclerosing cholangitis. Treatment with MF or VP was initiated at the age of 8 weeks, a point when the fibrotic phenotype is fully established. Mice were sacrificed at 20 weeks of age.

© 2023. Ye L et al. Published online at http://www.aginganddisease.org/EN/10.14336/AD.2023.0602

# SUPPLEMENTARY DATA

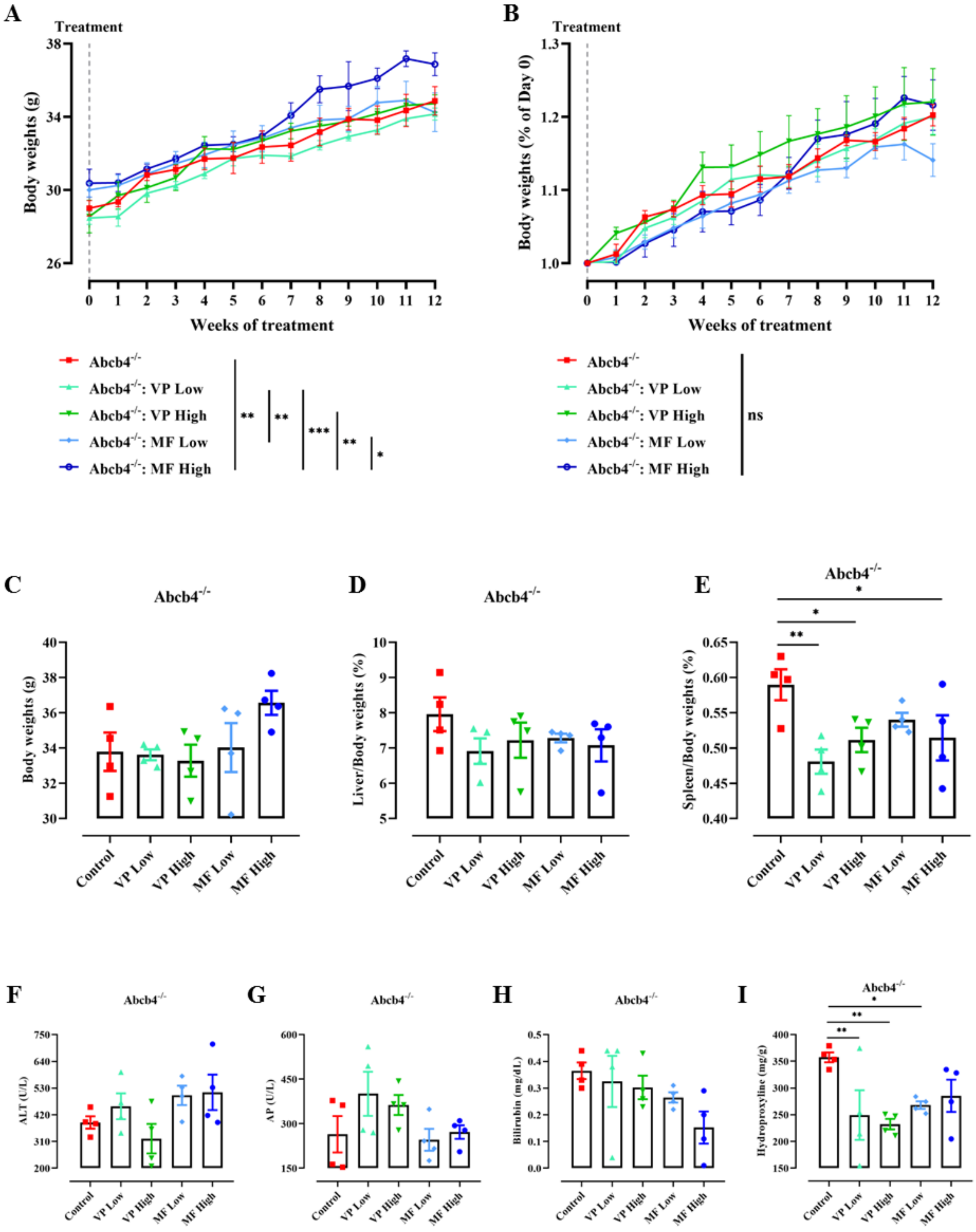


**Supplementary Figure 5. Effects of verteporfin and metformin on weights and serum parameters in the Abcb4$^{-/-}$ model.** The effects on liver damage and the side effect profiles of low and high dosages of verteporfin (VP) and metformin (MF) were evaluated in the Abcb4$^{-/-}$ model. **A.** Body weights (gram) were measured over the course of treatments and were normalized as ratios (%) with reference to their original weights since initiation **(B)**. At the end of the experiments, the body weights (gram) were measured **(C)**, and the ratios (%) of liver/body weights **(D)** and spleen/body weights **(E)** were calculated. Serum parameters of ALT (U/L) **(F)**, AP (U/L) **(G)**, and total bilirubin (mg/dL) **(H)** were measured under treatment conditions. **I.** Levels of liver hydroxyproline (mg/g) were measured. ns, not significant; ALT, alanine aminotransferase; AP, alkaline phosphatase. n=4. *, $P$<0.05, **, $P$<0.01, ***, $P$<0.001, ANOVA; test of normality by Shapiro-Wilk ($P$>0.05). The results are shown as mean ± standard error of the mean.

© 2023. Ye L et al. Published online at http://www.aginganddisease.org/EN/10.14336/AD.2023.0602

# SUPPLEMENTARY DATA

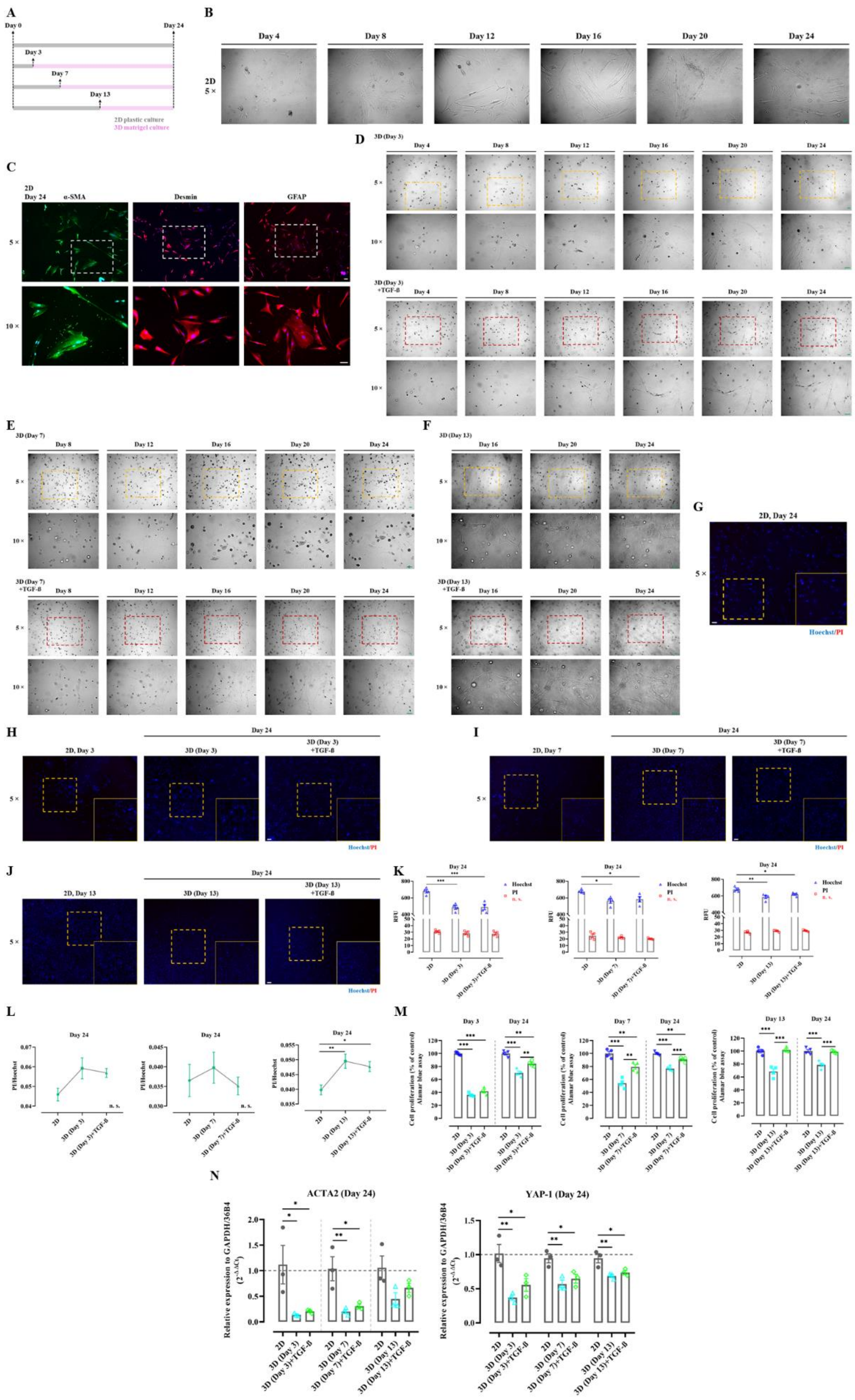

© 2023. Ye L et al. Published online at http://www.aginganddisease.org/EN/10.14336/AD.2023.0602

# SUPPLEMENTARY DATA

**Supplementary Figure 6. Proliferation and activation can be prevented by changing environmental stiffness from `hard" to `soft` surface. A.** The experimental design was illustrated. phHSC were transferred at different time points of activation {day 3: 3D (day 3); day 7: 3D (day 7); day 13: 3D (day 13)} from 2D plastic dishes to 3D matrigel cultures. **B.** A typical growing pattern of phHSC in 2D plastic culture until day 24 was illustrated by bright field images. **C.** Activation process was shown by immunofluorescence for α-SMA, desmin, and GFAP. Magnification, 5× and 10×, scale bar=50 µm. **D-F.** Sequential images of phHSC of all groups with or without TGF-β (10 ng/ml) stimulation were illustrated. Magnification, 5× and 10×, scale bar=50 µm. **G.** The image showed a Hoechst/PI cell death staining of phHSC in 2D culture on day 24. Magnification, 5×, scale bar=50 µm. **H-J.** Hoechst/PI staining of experimental groups with or without TGF-β were shown. Magnification, 5×, scale bar=50 µm. **K.** Relative fluorescence unit (RFU) of Hoechst (excitation 361 nm; emission 486 nm) and PI on day 24 of different experimental groups with or without TGF-β were calculated. n=4. **L.** The PI/Hoechst ratio as a marker of cell death was calculated, respectively. **M.** Proliferation of different experimental groups was measured by alamar blue assay. n=4. The values were normalized to the mean of the control group in percentage (%). **N.** Quantitative rt-PCR was performed to assess the expression of ACTA2 and YAP ($2^{-\Delta\Delta Ct}$) in phHSC. n=3. The values shown were normalized based on the mean of GAPDH and 36B4 in indicated control group. n. s., not significant. *, $P$<0.05, **, $P$<0.01, ***, $P$<0.001, ANOVA; test of normality by Shapiro-Wilk ($P$>0.05). The results are shown as mean ± standard error of the mean. The squares in dashed lines indicated the areas shown in 10-folds images.

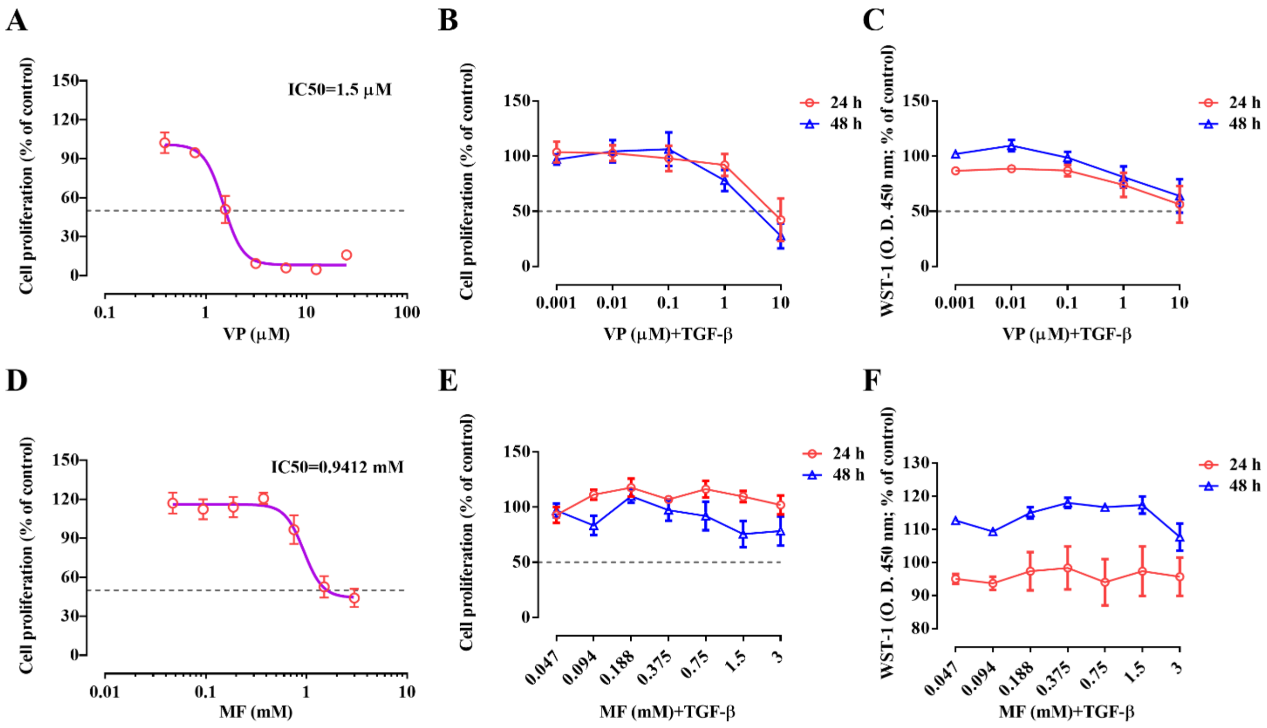


**Supplementary Figure 7. Assessment of cell proliferation and viability under verteporfin or metformin treatment in LX-2 cells.** Proliferation was assessed by Sybr green assay and viability was measured by WST-1 assay. Quantification was normalized to control-treated (vehicle) cells under indicated concentrations of verteporfin (VP) or metformin (MF) with or without TGF-β (10 ng/ml). **A-B.** Proliferation under VP treatment was assessed in LX-2 cells with or without TGF-β. n=3. **C.** WST-1 assay under treatment with VP was assessed in TGF-β activated LX-2 cells. n=3. **D-E.** Proliferation of LX-2 cells under MF treatment with or without TGF-β was assessed. n=3. **F.** MF treatment was assessed in TGF-β activated LX-2 cells by WST-1 assay. n=3. These values were normalized to the means of indicated control groups in percentage (%). IC50, half-maximal inhibitory concentration. O. D., optical density. The results are shown as mean ± standard error of the mean.

© 2023. Ye L et al. Published online at http://www.aginganddisease.org/EN/10.14336/AD.2023.0602
